# Host-microbiome-immune disequilibrium in oral disease: mechanisms, dysbiosis, and precision therapeutics

**DOI:** 10.3389/fimmu.2026.1854213

**Published:** 2026-06-22

**Authors:** Ming Lv, Wenya Xu, Tong Wang, Kehao Mou, Zijian Ni, Qiudi Tu, Jingkun Zhang, Xue Wu, Siyuan Song, Gang Cheng

**Affiliations:** 1 Department of Stomatology, The First People’s Hospital of Lin’an District, Hangzhou, Zhejiang, China; 2 Department of Emergency Medicine, Hangzhou Lin’an Traditional Chinese Medicine Hospital, Hangzhou, Zhejiang, China; 3 Department of Biology, Duke University, Durham, NC, United States; 4 School of Stomatology, Hangzhou Normal University, Hangzhou, Zhejiang, China; 5 School of Stomatology, Zhejiang Chinese Medical University, Hangzhou, Zhejiang, China; 6 Urology & Nephrology Center, Department of Nephrology, Zhejiang Provincial People’s Hospital, Affiliated People’s Hospital, Hangzhou Medical College, Hangzhou, Zhejiang, China; 7 Department of Physiology, University of California, San Francisco, San Francisco, CA, United States; 8 Institute for Global Health Sciences, University of California, San Francisco, San Francisco, CA, United States; 9 Department of Neuroscience, Baylor College of Medicine, Houston, TX, United States; 10 Department of Anesthesiology, Montefiore Medical Center, Albert Einstein College of Medicine, Bronx, NY, United States; 11 Center for Plastic & Reconstructive Surgery, Department of Stomatology, Zhejiang Provincial People’s Hospital, Hangzhou Medical College, Hangzhou, Zhejiang, China

**Keywords:** dysbiosis, host-microbiome interactions, immune homeostasis, oral microbiome, oral mucosal immunity, precision oral medicine

## Abstract

**Background:**

The oral cavity harbors a dynamic microbial ecosystem that interacts with epithelial barriers, host immunity, and local tissue environments. Disruption of this balance is increasingly recognized as a key driver of major oral diseases, including periodontitis, dental caries, and oral squamous cell carcinoma (OSCC). However, the biological links between microbial ecology, immune regulation, and disease progression are insufficiently integrated, limiting mechanistic understanding and translational progress.

**Methods:**

This structured narrative review searched PubMed/MEDLINE, Web of Science, Embase, and Scopus for relevant studies on oral microbiome ecology, mucosal immunity, dysbiosis, oral diseases, and emerging therapies. Evidence was narratively synthesized across microbiome ecology, mucosal immunology, disease pathogenesis, and translational research, with consideration of study type, mechanistic relevance, and translational significance.

**Results:**

Current evidence supports that oral homeostasis relies on coordinated interactions among commensal microbial communities (CMC), epithelial and salivary barriers, and immune surveillance. Dysbiosis disrupts this equilibrium by promoting the expansion of pathobionts, amplifying inflammatory responses, and contributing to tissue injury. This systems-level perspective helps explain the persistence and heterogeneity of oral diseases beyond pathogen-centered models. Emerging technologies are reshaping this field. These include microbiome-modulating therapies, host-directed interventions, multi-omics approaches, and artificial intelligence (AI). These approaches are advancing disease stratification, biomarker discovery, and precision therapeutic development.

**Conclusion:**

Oral diseases should be understood as disorders of host-microbiome-immune disequilibrium rather than as isolated infections. This perspective highlights the need for integrated strategies that consider microbial ecology, immune regulation, epithelial barrier function, and clinical context to improve prevention, diagnosis, and treatment in precision oral medicine.

## Graphical Abstract

**Figure:**
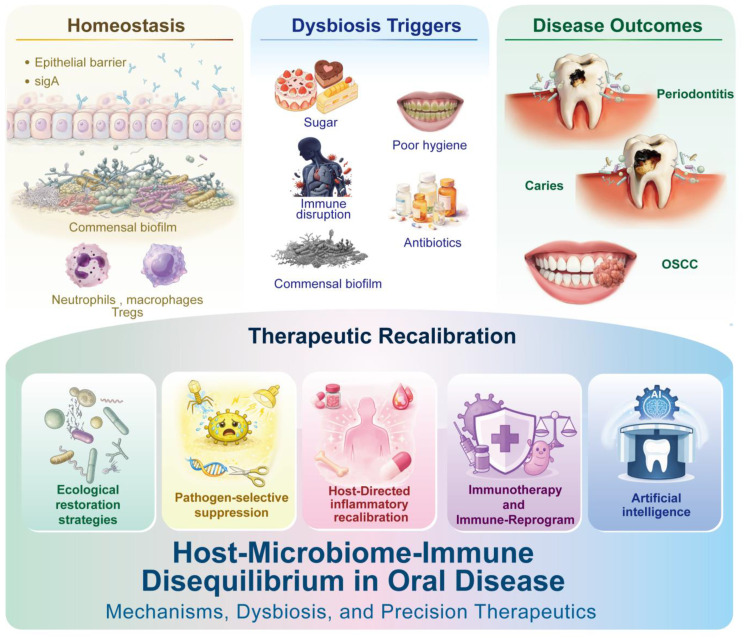

## 1 Introduction

The oral cavity contains one of the most diverse and densely inhabited microbial ecosystems in the human body, with bacteria, fungi, viruses, and archaea living in specialized ecological niches, such as the teeth, tongue, gingiva, and saliva (1, 2). The oral microbiome is not merely a passive microbial reservoir, but a highly organized ecological system that contributes to colonization resistance, nutrient metabolism, epithelial barrier integrity, and immune education (3). Oral epithelial cells form a multilayered barrier and produce antimicrobial peptides (AMPs) and cytokines that restrict microbial invasion (4, 5). In parallel, innate and adaptive immune cells continuously monitor microbial communities (6). Oral tissue macrophages participate in inflammatory regulation and tissue repair, thereby shaping cytokine balance and wound-healing responses (7). Dendritic cells (DCs) coordinate antigen-specific immune responses, whereas salivary secretory Immunoglobulin A (sIgA) limits microbial adhesion to mucosal surfaces (3). These components collectively create a meticulously regulated interaction between host defense and microbial ecology.

Oral homeostasis is sustained through the coordinated interplay of epithelial barriers, salivary defenses, immunological surveillance, and CMC. Commensal bacteria have an active role in maintaining the stability of mucosal membranes by supporting epithelial antimicrobial systems (8). Disruption of this balance promotes ecological instability and dysregulated host responses (9, 10), contributing to oral diseases such as dental caries, periodontitis, and OSCC (11).

Traditional models of oral disease focus on specific microorganisms but often overlook the more complex factors that contribute to the development of the disease. Disease progression is more appropriately viewed as a consequence of ecological disruption and dysregulated host responses rather than as a single-pathogen infection (12). Environmental and behavioral factors, including nutrition, dental hygiene, and smoking, can modify microbial composition, alter local metabolic conditions, and elevate inflammatory signals (10, 13). Although these interactions are increasingly recognized, existing evidence remains fragmented. Taxonomic descriptions generally come before mechanistic integration, biological interpretation, and translational application.

This review fills in these gaps by bringing together the most up-to-date information on oral homeostasis, dysbiosis, and disease under a single framework for the host, microbiome, and immune system. We propose that oral diseases should be understood as context-dependent failures of this integrated host-microbiome-immune system. We also stress the importance of systems biology and multi-omics integration in order to better understand how diseases progress. This is because pathogen-centered models do not fully capture the complexity of host-microbiome-immune interactions. We delve into novel therapeutic techniques and examine how multi-omics integration, AI, and microbiome-targeted interventions can transform the area from descriptive profiling to mechanism-informed, clinically actionable precision oral medicine.

### 1.1 Review design and literature selection

This manuscript was designed as a structured review. To improve methodological transparency, we conducted targeted literature searches in PubMed/MEDLINE, Web of Science, Embase, and Scopus. Search terms included combinations of “oral microbiome,” “dental biofilm,” “mucosal immunity,” “host-microbe interactions,” “dysbiosis,” “periodontitis,” “dental caries,” “OSCC,” “probiotics,” “host-modulatory therapy,” “immunotherapy,” “multi-omics,” and “artificial intelligence.” Reference lists of key reviews and primary studies were also screened to identify additional relevant publications.

Studies were considered eligible if they provided mechanistic, ecological, clinical, or translational evidence related to oral host-microbiome-immune interactions. We prioritized systematic reviews and meta-analyses, randomized or controlled human studies, representative observational microbiome studies, animal models, and *in vitro* mechanistic studies directly relevant to oral homeostasis, dysbiosis, disease pathogenesis, or therapeutic intervention. Duplicate articles, conference abstracts without full text, studies not focused on oral disease biology, and articles lacking sufficient methodological or mechanistic information were excluded. Evidence was critically interpreted according to study design, biological plausibility, clinical relevance, and translational limitations.

Oral diseases arise from disruption of host-microbiome-immune homeostasis, driven by environmental and microbial factors, and can be counteracted by integrated therapeutic strategies.

## 2 Ecological organization and functional composition of the oral microbiome

**Figure 1:**
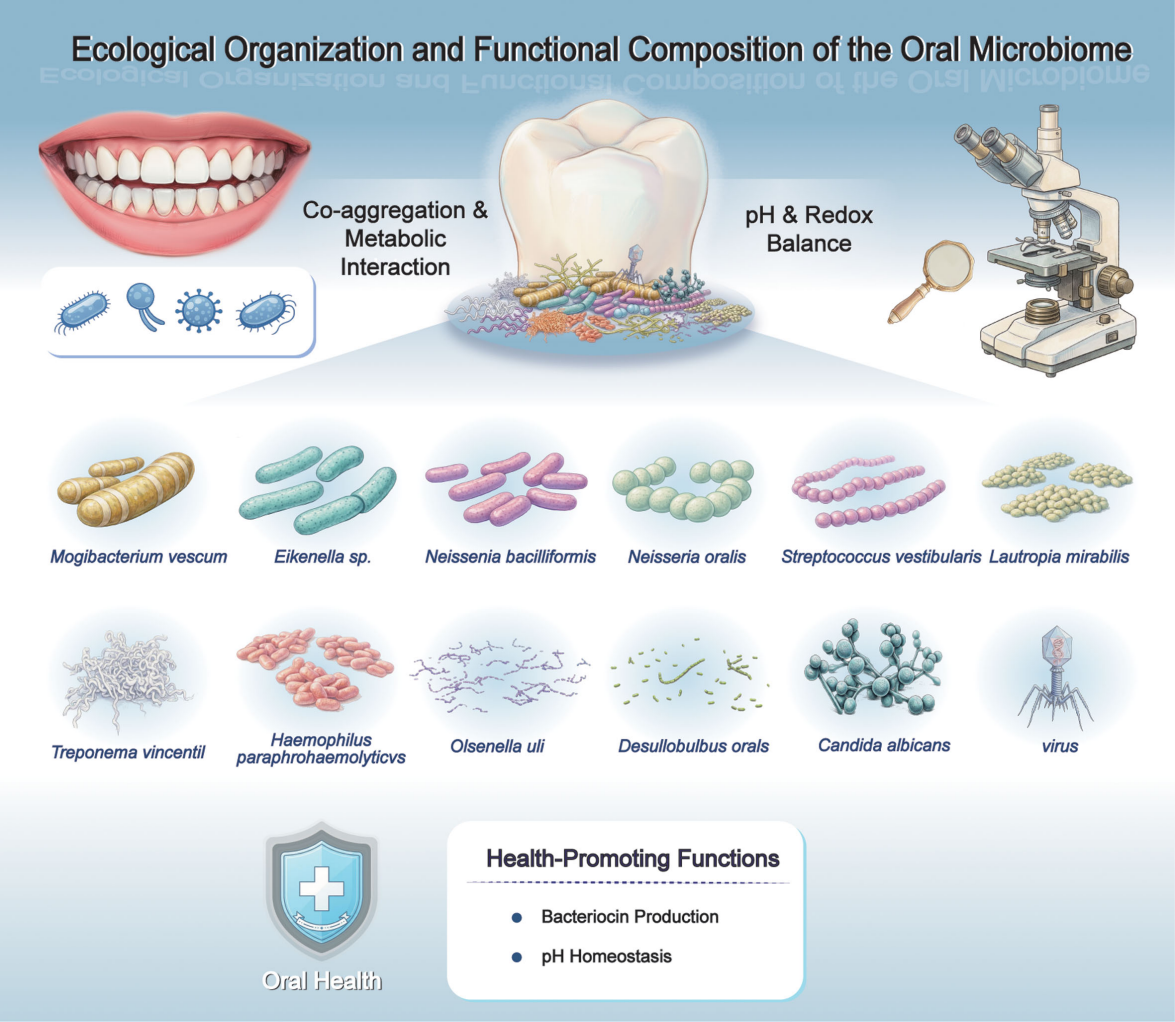
Ecological architecture of the healthy oral microbiome. The oral microbiome is structured into biofilms on oral surfaces, where microbial interactions such as co-aggregation and metabolic exchange support health functions like pH balance and bacteriocin production. These interactions maintain oral health, with disruptions leading to dysbiosis.

The oral microbiome includes bacteria, fungi, viruses, and archaea. Dominant bacterial phyla include *Firmicutes*, *Bacteroidetes*, *Actinobacteria*, *Proteobacteria*, *Fusobacteria*, and *Spirochaetes* (14). Under healthy conditions, commonly observed genera include *Streptococcus* (e.g. *S. sanguinis*, *S. salivarius*), *Actinomyces*, *Veillonella*, *Neisseria*, and *Corynebacterium* (1, 15). The oral cavity contains not only bacteria, but also fungi, salivary viruses, and *methanogenic archaea*, highlighting the oral microbiome as a biologically interconnected ecosystem. Salivary viruses encompass bacteriophages that can alter the architecture of bacterial communities, alongside human viruses such as herpesviruses and papillomaviruses, which frequently present with no symptoms (16). This multi-domain composition constitutes the ecological foundation for the establishment of oral homeostasis and susceptibility to illness (**Figure 1**).

Oral bacteria form biofilms on the teeth and mucosal surfaces. Biofilms are stabilized by microbial adhesion, co-aggregation, and metabolic exchange. Oral biofilms are spatially organized microbial communities shaped by interspecies interactions and local environmental conditions. Some bacteria help the biofilm grow by physically holding different organisms together (17). Some organisms engage in metabolic cross-feeding, using metabolites produced by one species as substrates for another. In this ecological context, taxa such as *Veillonella* and *Neisseria* can modulate local environmental conditions through lactate or nitrate metabolism, thereby influencing pH balance, redox potential, and health-associated biofilm stability. Thus, oral biofilms are dynamic, spatially structured microbial communities, not surface deposits.

The oral microbiome supports ecological and immunological equilibrium. Commensal species, including *S. sanguinis* and *S. salivarius*, contribute to this equilibrium by generating bacteriocins and alkaline metabolites via arginine and urea metabolism, thus protect enamel and inhibit pathogenic colonization (18, 19). In general, beneficial oral bacteria prevent pathobionts, resist colonization, and maintain pH and redox levels (20, 21). They keep the mucosal environment healthy and teach the immune system how to work properly. When interactions are adequately controlled, the mouth has a stable cooperative ecology. Diseases of the mouth and body can happen when the environment is out of balance (22). These data suggest oral health depends on host-microbiome equilibrium.

To provide a concise overview of the major microbial taxa in oral homeostasis, **Table 1** summarizes representative normal oral microorganisms.

**Table 1:** Representative members of the normal oral microbiome and their associated conditions.

Category	Microorganism/taxon	Body site	Ecological roles	Functional role in healthy oral environment	Reference
Bacteria	Mogibacterium vescum	Oral	As a part of the normal oral microbiota.	Low-abundance anaerobic Gram-positive oral resident; participate in peptide-rich anaerobic biofilm metabolism and metabolic cross-feeding within mature plaque communities.	HOMD (23);
	Eikenella sp./Eikenella corrodens	Oral	As a part of the normal oral microbiota and is commonly found in the oral cavity.	Participates in multispecies biofilm organization through lectin-like coaggregation with other oral bacteria; facultative anaerobic metabolism may help it occupy oxygen-gradient interfaces in dental plaque.	HOMD (24);
	Neisseria bacilliformis	Oral	A cultivated member of the human oral microbiota listed in HOMD as oral HMT-013. It occurs as part of oral microbial communities	Member of oral Neisseriaceae; may participate in aerobic/facultative biofilm metabolism and oxygen/redox-associated niche organization. Nitrate-related metabolic functions are better supported for oral Neisseria spp./Neisseriaceae at the genus or family level rather than specifically for N. bacilliformis.	HOMD (25, 26);
	Neisseria oralis	Oral	A cultivated oral Neisseria species and member of the human oral microbiota, detected in subgingival and supragingival plaque, buccal mucosa, saliva, and other oral sites, including healthy subjects.	Oral Neisseria species with potential nitrate-reducing activity; may help convert nitrate to nitrite, support pH stability in dental plaque, and limit overgrowth of acid-producing or anaerobic disease-associated bacteria. Evidence is mainly genus-level, with emerging support for N. oralis in plaque nitrate reduction.	HOMD (26, 27);
	Streptococcus vestibularis	Oral	A cultivated oral Streptococcus species and common member of the human oral microbiota. It was originally isolated mainly from the vestibular mucosa of human oral cavities and is detected across healthy oral sites, including buccal mucosa, hard palate, saliva, tongue dorsum, and supra-/subgingival plaque.	Salivarius-group oral commensal; may support oral homeostasis through urease-related ammonia production and hydrogen peroxide production. Host salivary factors, including ZG16B and MUC7, can bind S. vestibularis and promote bacterial aggregation, which may contribute to mucin-assisted microbial clearance and mucosal homeostasis.	HOMD (28, 29);
	Lautropia mirabilis	Oral	A cultivated, high-abundance oral taxon listed in HOMD and commonly detected in dental plaque and gingival biofilm communities.	Facultative anaerobic, urease-positive, nitrate/nitrite-reducing plaque organism; may support local pH buffering, nitrate-related redox metabolism, and adaptation to gingival biofilm niches.	HOMD (30, 31);
	Treponema vincentii	Oral	Oral anaerobic Treponema species detected in subgingival plaque.	Motile oral spirochete that can inhabit anaerobic subgingival biofilm layers. It may participate in proteolytic anaerobic community interactions and syntrophic relationships with other periodontal taxa.	HOMD (31, 32);
	Haemophilus paraphrohaemolyticus	Oral/Upper respiratory tract	A cultivated Haemophilus species listed in HOMD as an oral HMT-035 taxon and detected across multiple oral sites in healthy-subject datasets.	Facultative/microaerophilic Pasteurellaceae member; may contribute to site-specific oral biofilm ecology, local metabolic interactions, and adaptation to oxygen-variable plaque or mucosal environments	HOMD (33);
	Olsenella uli	Oral	A cultivated oral Olsenella species listed in HOMD as HMT-038 and originally characterized from human oral isolates. It can be described as a low-abundance oral anaerobic resident.	Obligate anaerobic, Gram-positive oral taxon; may participate in carbohydrate fermentation and anaerobic metabolic interactions.	HOMD (34);
	Desulfobulbus oralis	Oral	A human oral/subgingival species and sulfate-reducing bacterium adapted to the periodontal niche.	Anaerobic sulfate reducer involved in sulfur cycling, redox metabolism, and sulfide production in subgingival plaque biofilms.	HOMD (35);
Fungi	Candida albicans	Oral	A common oral fungal colonizer that can exist as a low-level/asymptomatic commensal in healthy individuals. It usually coexists with oral bacteria and host mucosal defenses without causing disease	Low-abundance/asymptomatic oral mycobiome member; interacts with oral bacteria in mixed bacterial-fungal biofilms. Its commensal state is controlled by salivary factors, epithelial barriers, oral microbiota, and antifungal immune surveillance, which limit fungal overgrowth, hyphal invasion, and mucosal damage.	HOMD (36–38);
Viruses	Fusobacteriaceae_phage	Oral	Present in the healthy oral virome, these phages may influence bacterial community stability by interacting with bacteria hosts.	Regulate Fusobacterium populations through lytic or lysogenic infection, influencing bridging organisms involved in multispecies.	(39, 40)
	Streptococcaceae_phage	Oral	Present in the healthy oral virome, these phages may influence bacterial community stability by interacting with bacteria hosts.	May influence streptococcal strain diversity and abundance through lysogenic or lytic interactions. Phage-Streptococcus interactions may indirectly affect early biofilm and community stability.	(41, 42)
	Neisseriaceae_phage	Oral	Present in the healthy oral virome, these phages may influence bacterial community stability by interacting with bacteria hosts.	Shape Neisseria community structure through host-specific predation and lysogeny, potentially affecting nitrate-reducing community functions.	(42–45)
	Veillonellaceae_phage	Oral	Present in the healthy oral virome, these phages may influence bacterial community stability by interacting with bacteria hosts.	Regulate lactate-utilizing Veillonella populations, thereby indirectly influencing lactate cross-feeding and local acid/base balance in plaque	(44, 46)
	Pasteurellaceae_phage	Oral/pharynx/upper respiratory tract	Present in the healthy oral virome, these phages may influence bacterial community stability by interacting with bacteria hosts.	Regulate Pasteurellaceae members such as Haemophilus through phage predation or lysogenic conversion. Because these phages show niche specificity across oral sites, they may contribute to site-specific bacterial community turnover.	(25, 47)
	Prevotellaceae_phage	Oral	Present in the healthy oral virome, these phages may influence bacterial community stability by interacting with bacteria hosts.	May influence Prevotella abundance and strain-level dynamics through phage-host interactions and lysogeny. Because Prevotella species are anaerobic fermenters and can expand during periodontal dysbiosis, these phages may indirectly affect anaerobic biofilm remodeling.	(48, 49)
	Peptostreptococcaceae_phage	Oral/Gut	Detected in oral and gut phageome/metagenomic studies, these putative phages may interact with anaerobic Gram-positive bacterial hosts.	May influence Peptostreptococcaceae-related anaerobic populations through phage-host interactions or lysogeny, potentially affecting mature anaerobic biofilm ecology and peptide/amino-acid-rich metabolism.	(44, 50)
Archaea	Methanogens	Oral	Representative methanogenic archaea detectable in oral biofilms, including periodontally healthy individuals.	Consume H_2_ and CO_2_ to produce methane, potentially lowering hydrogen pressure and supporting anaerobic syntrophy with fermentative bacteria in mature subgingival biofilms. Their contribution should be interpreted as context-dependent ecological metabolism rather than a proven protective function.	(51, 52)

HOMD, Data adapted from the Human Oral Microbiome Database (https://homd.org/).

## 3 Immune regulation in the oral cavity

### 3.1 Innate immunity

The oral mucosa serves as a permeable barrier that maintains a delicate balance with microbiota, supported by organized immune structures that expand during inflammation (53). This barrier is not only physical but also immune-responsive, with epithelial cells expressing pattern recognition receptors (PRRs), such as Toll-like receptors (TLRs), to detect microbial signals and initiate inflammatory responses. When disrupted by pathogenic bacteria, this barrier-immune interface can contribute to tissue damage and potentially cancer-related inflammation (54). However, commensal bacteria contribute to homeostasis by inducing epithelial innate responses, including β-defensin expression, thereby maintaining a pathogen-resistant epithelial state (55).

Saliva, as an indicator of the oral environment, harbors various bacteria and immune cells (56). Interleukin-8 (IL-8) gradients recruit neutrophils, the predominant immune cells in saliva, to the gingival crevice. They make up roughly 30-40% of salivary cells and more than 95% of leukocytes. As critical innate immune effectors, neutrophils restrict pathogenic colonization through phagocytosis, protease release (e.g., elastase, Myeloperoxidase (MPO)), oxidative burst (Reactive Oxygen Species (ROS)), and the formation of neutrophil extracellular traps (NETs). Their importance in dental health is evident from the early onset of periodontitis in congenital neutrophil anomalies, such as leukocyte adhesion deficiency (6). In periodontitis, neutrophil hyperactivation can establish a self-reinforcing cycle of inflammation, dysbiosis, and tissue-destructive immune responses. This highlights the importance of modulating host inflammatory responses in periodontal therapy (57).

Macrophages and DCs are also central components of mucosal immunity. Macrophages, controlled by Triggering Receptor Expressed on Myeloid Cells 2 (TREM2), are essential in oral inflammation and tissue remodeling. TREM2^+^ macrophages display immunoregulatory roles, evidenced in periodontal disease (PD), where an increased M1/M2 ratio indicates a pro-inflammatory condition (58). Macrophages are key controllers of inflammation in the mouth and tissue remodeling. In periodontitis (PD), oral leukoplakia (OL), oral lichen planus (OLP), and OSCC, there has been an increase in macrophage infiltration and M2 polarization. This is especially true for OLP and metastatic OSCC, and it alters over time with anti-PD1 treatment (59). In PD, a higher M1/M2 ratio is another sign of a pro-inflammatory condition. DCs, such as Langerhans cells, connect innate and adaptive immunity by taking samples of microbial antigens and showing them to CD4+ and CD8+ T cells (60, 61). Lysozyme, lactoferrin, histatins, and peroxidases are salivary innate immune effectors that inhibit a broad range of microorganisms (3, 62).

These innate immune layers cooperate to maintain biofilm homeostasis by discriminating between commensal microorganisms and potential threats. This helps prevent microbial dysbiosis under healthy oral conditions.

### 3.2 Adaptive immunity

Adaptive immunity in the oral mucosa provides long-lasting protection while maintaining tolerance to commensal microbiota and food antigens.

#### 3.2.1 T lymphocytes

The immune-active oral mucosa must balance protection and tolerance. When this balance is off, OLP and gingivitis occur. T Helper 17 Cells (Th17) cells bring neutrophils to the mucosa, boosting immunity. However, excessive Th17/Interleukin-17 (IL-17) responses may cause immunopathology (63). Oral epithelial cells are important for immunological tolerance because they change both innate and adaptive immune responses (64). This prevents the immune system from going too far when it fights infections and protects innocuous commensals.

#### 3.2.2 B cells and plasma cells

The major antibody in saliva and other mucosal secretions is sIgA produced by B and plasma cells in oral-associated lymphoid tissues and salivary glands. Mucosal immunity relies on sIgA binding to toxins and microbial antigens, clumping microorganisms, and preventing them from attaching to epithelial cells. This limit infections and maintains helpful microorganisms (65). Immunoglobulin G (IgG) is found in saliva and mostly comes from the blood. Secretory sIgA, on the other hand, is generated in the mouth and conveyed via a particular receptor. Stress or blood contamination can alter antibody levels in saliva; yet saliva remains a valuable fluid for examining immunological health and the immune system’s modifications during sickness (66).

**Figure 2:**
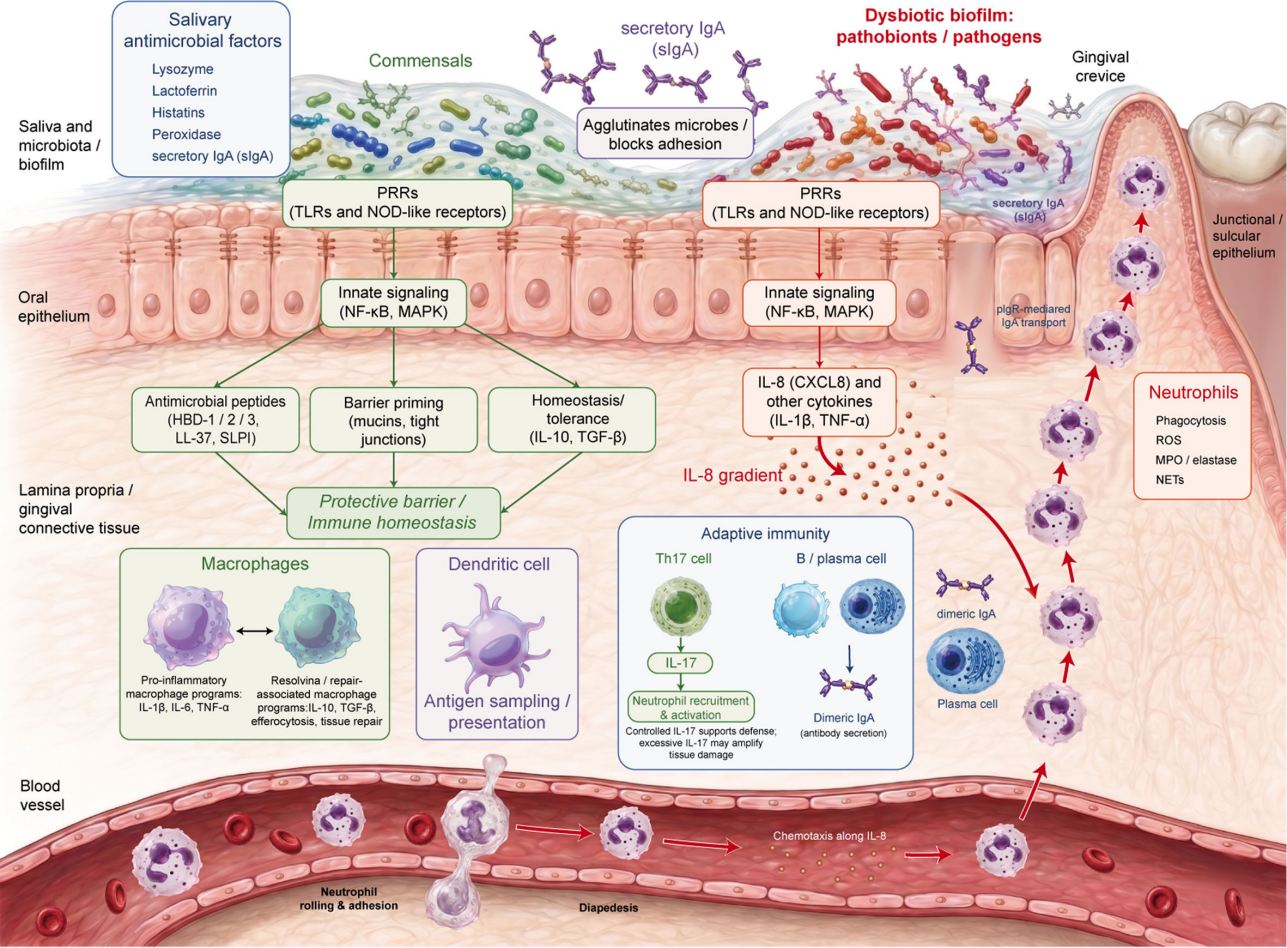
Host-microbiome immune interactions in the oral cavity. This schematic illustrates the balance between oral immune homeostasis and dysbiotic inflammation. In health, salivary antimicrobial factors and secretory IgA limit microbial adhesion, while commensal sensing through epithelial PRRs/TLRs/NOD-like receptors induces controlled NF-κB/MAPK signaling, antimicrobial peptides, barrier reinforcement, and regulatory mediators such as IL-10 and TGF-β. These responses support epithelial integrity, immune tolerance, macrophage-mediated resolution, dendritic-cell antigen sampling, Th17-mediated mucosal defense, and IgA secretion. Under dysbiotic conditions, pathobiont-enriched biofilms trigger excessive epithelial inflammatory signaling, including IL-8/CXCL8, IL-1β, and TNF-α production. The resulting IL-8 gradient recruits neutrophils from the bloodstream to the gingival crevice, where phagocytosis, ROS, MPO, elastase, and NETs contribute to microbial clearance but may also amplify tissue inflammation when persistent.

These coordinated innate and adaptive immune processes maintain oral homeostasis and are summarized in **Figure 2**.

## 4 Dysbiosis and disease pathogenesis

Disruptions in the host-oral-microbiome equilibrium can lead to dysbiosis, a context-dependent ecological state involving changes in microbial abundance, community structure, metabolic activity, and host immune compatibility. Environmental and behavioral factors, such as high-sugar diets, smoking, and antibiotic use, may further perturb this equilibrium (10). In this context, dysbiosis may create ecological and immunological conditions that favor the expansion of pathobionts, altered microbial metabolism, and dysregulated host responses, thereby compromising oral homeostasis. Protective commensal functions that support ecological stability and colonization resistance may be weakened, while pathobionts may promote inflammation or reshape local metabolic conditions (67, 68). Therefore, major oral diseases reflect host-microbiome-immune disruption rather than single-pathogen infection.

The shift from oral health to dysbiosis is driven by changes in the local ecological niche. Different factors create selective pressures: frequent sugar intake lowers plaque pH and favors acid-tolerant bacteria; plaque accumulation, smoking, and inflammation promote anaerobic and proteolytic communities in the gingival crevice; and tumor-related hypoxia and epithelial disruption can reshape the microbiome in OSCC. In each case, these environmental and host-related factors do more than alter microbial composition—they actively select for communities that amplify inflammation, disrupt metabolism, and contribute to tissue damage. This provides a mechanistic link explaining why different oral diseases are associated with distinct patterns of dysbiosis.

### 4.1 Periodontitis: inflammation-stabilized dysbiosis

Periodontitis is a chronic inflammatory disease affecting the tooth-supporting tissues. It is a clear case of inflammophilic dysbiosis, in which inflammation actually helps keep the microbial imbalance in place. Clinically, periodontitis frequently develops from gingivitis brought on by plaque to attachment loss, alveolar bone resorption, and ultimately tooth loss (69, 70). The defining feature of periodontitis is not merely the presence of periodontal pathogens, but the establishment of a self-reinforcing inflammatory cycle. In this cycle, the body’s inflammation changes the local environment, which allows pathobionts to grow and associated with even more inflammation, leading to further damage to the tissues.

This inflammatory periodontal niche favors the growth of a dysbiotic subgingival community, enriched in species such as *Porphyromonas gingivalis*, *Tannerella forsythia*, *Treponema denticola*, and *Fusobacterium nucleatum*. These species not only thrive in inflammatory environments but also contribute to the persistence and amplification of inflammation. *Methanogenic archae*a, especially *Methanobrevibacter oralis*, are often found in subgingival biofilms and have been associated with the severity of periodontitis, although their exact function is not fully elucidated (71). Among these organisms, *P. gingivalis* is often regarded as a keystone pathogen because it can reshape the microbial ecosystem and exacerbate inflammation through virulence factors such as lipopolysaccharide (LPS) and proteases, thereby perpetuating dysbiosis and chronic inflammation (72).

This inflammatory niche is often generated by plaque accumulation, impaired oral hygiene, smoking, or systemic inflammatory susceptibility. As the subgingival biofilm matures, oxygen availability decreases and the gingival crevice becomes enriched with inflammatory exudates, heme-containing compounds, peptides, and tissue-breakdown products (57). These nutrient and redox changes selectively favor proteolytic and inflammophilic anaerobes, including *P. gingivalis*, *T. forsythia*, *T. denticola*, and *F. nucleatum*, while weakening health-associated colonization resistance. In parallel, neutrophil hyperactivation, NET formation, macrophage polarization, Interleukin-1 Beta (IL-1β), Interleukin-6 (IL-6), Tumor Necrosis Factor Alpha (TNF-α), IL-17, MMPs, and Receptor Activator of Nuclear Factor Kappa-B Ligand (RANKL) promote connective tissue degradation and osteoclast-mediated bone resorption (6, 58). Tissue destruction then releases additional inflammatory nutrients, creating a feed-forward loop in which host inflammation becomes an ecological driver of microbial dysbiosis.

Periodontitis reflects a self-reinforcing interaction between microbial dysbiosis and immune-mediated tissue damage. Host inflammation reshapes the local periodontal environment, favoring the expansion of pathobionts and further amplifying inflammatory tissue injury. As connective tissue breakdown progresses, tissue-derived nutrients and inflammatory mediators help sustain this dysbiotic cycle. Periodontitis also represents an important local-systemic interface. During daily oral activities or dental procedures, periodontal pathogens and inflammatory mediators may enter the bloodstream, providing a potential link between localized periodontal inflammation and systemic inflammatory conditions, including cardiovascular disease, diabetes, respiratory disease, rheumatoid arthritis, chronic kidney disease, neurodegeneration, adverse pregnancy outcomes, and some cancers (73–76). Consequently, periodontitis is more precisely characterized as a chronic inflammatory environment in which microbial dysbiosis, host injury, and systemic inflammatory consequences are interconnected.

### 4.2 Dental caries: metabolic acidification and ecological collapse

Dental caries have acid-induced oral imbalance. Sugary food consumption alters the mouth’s flora, weakening our teeth’s protective covering. While cariogenic bacteria including *mutans streptococci*, *lactobacilli*, *Scardovia wiggsiae*, and several *Actinomyces species* are significantly linked to caries, the condition cannot be completely elucidated by any singular organism. Regular sugar intake can contribute to acid buildup, altering the mouth’s microbial balance and causing caries.

Eating sugar encourages bacteria to create acid, which lowers plaque pH and damages dental enamel and dentin, favoring aciduric species. *Streptococcus mutans* may turn sucrose into lactic acid and help cariogenic biofilm grow (77, 78). However, its role is not best understood as that of an isolated pathogen; rather, it contributes to the establishment and maintenance of an acid-dominated ecological state.

Frequent consumption of fermentable carbohydrates drives repeated acidification in dental plaque, lowering pH and favoring the growth of aciduric and acidogenic taxa. This ecological pressure selects for cariogenic bacteria, including *S. mutans*, *Lactobacillus* spp., *Actinomyces* spp., *Scardovia/Bifidobacterium* spp., and *Candida albicans*, which collectively form stable, EPS-rich biofilms. As the biofilm gets more acidic over time, the microbial community that can live in low-pH environments. As lesions get worse, *S. mutans* makes more glucan-binding adhesins and extracellular polysaccharides, which makes plaque build-up and the structure stay stable (79). Thus, caries is resulting from ecological selection under metabolic stress.

This framework suggests that caries prevention should not only suppress cariogenic microorganisms, but also restore a less acidogenic oral environment through dietary control, enamel protection, and maintenance of microbial ecological balance (80).

### 4.3 OSCC: tumor-associated microbial remodeling

Emerging evidence links oral microbiome dysbiosis to OSCC, a major subtype of head and neck cancer. The microbiome’s participation in OSCC, in contrast to periodontitis and caries, is not optimally characterized as a traditional infectious process. OSCC is more accurately characterized as a disease influenced by microbial remodeling and tumor-promoting inflammatory interactions, wherein altered epithelial states, chronic inflammation, and modifications in microbial communities become increasingly interconnected.

A key part of this process is how microbes change the environment around the tumor. OSCC tissues and saliva often contain high levels of bacteria linked to gum disease and those that thrive without oxygen, particularly *Fusobacterium nucleatum* and *Porphyromonas gingivalis*, while typical commensals are relatively depleted (81, 82). These changes suggest that the tumor environment encourages the growth of microbes that thrive in places with inflammation, low oxygen, or metabolic problems.

Tumor-promoting inflammation is increasingly recognized as an important component of OSCC pathogenesis. Persistent inflammation contributes to a permissive tumor microenvironment by modulating immune surveillance, enhancing pro-inflammatory signaling, and altering saliva-mediated host-microbiome interactions (83). In this case, dysbiosis create a tumor-permissive microenvironment that supports tumor growth, immune evasion, and invasion.

Microbe-epithelium oncogenic signaling is an important component of OSCC-associated microbial remodeling. *F. nucleatum* may promote epithelial proliferation, invasion, and immune evasion, partly through β-catenin activation and inflammatory signaling pathways (84, 85). *P. gingivalis* can invade epithelial cells and modulate IL-6/Signal Transducer and Activator of Transcription 3 (STAT3), NF-κB, and anti-apoptotic signaling, thereby contributing to a pro-inflammatory and tumor-permissive microenvironment (86, 87). Fungal and viral factors may further participate in microbial and inflammatory remodeling in OSCC. For example, *Candida albicans* may exacerbate epithelial injury and carcinogenic stress through hyphal invasion, inflammatory activation, and acetaldehyde production, while human *papillomavirus* may interact with epithelial and immune alterations in selected OSCC contexts (88–91). Together, these mechanisms do not support a simple single-pathogen model but rather suggest that OSCC-associated dysbiosis emerges from reciprocal interactions among epithelial transformation, immune dysregulation, and microbial functional adaptation.

These results corroborate the notion that the microbial dysbiosis in OSCC is attributable to a multifaceted interplay of tissue alterations, inflammation, and variations in the microbial milieu, rather than being induced by a singular organism.

### 4.4 Oral dysbiosis beyond the oral cavity

Oral bacteria imbalances associated with long-term inflammation that affects mouth and health. Oral microorganisms can circulate and impact the heart, gut, and brain. This can contribute to the systemic inflammatory responses. There is more and more proof that the mouth is a physiologically active systemic inflammatory interface that can affect distant organs and physiological systems through microbial products, inflammatory mediators, and dysregulated host responses (92). Cardiometabolic illness is one of the most researched areas. Periodontal bacteria can enter the circulation and travel to distant tissues, including the blood vessels, where they participate in processes linked to endothelial dysfunction, inflammation of the blood vessels, and the formation of plaques (93, 94). Chronic oral inflammation is linked to increased systemic inflammatory markers, including C-reactive protein (CRP), insulin resistance, and type 2 diabetes (95).

The gut and gastrointestinal axis is another significant area. Oral bacteria and their metabolites may modify the makeup of gut microbiota and mucosal immunological systems, hence leading to inflammatory bowel disease (IBD) and potentially colorectal cancer (96). A third new area of research is neuroinflammatory and neurodegenerative diseases. Oral microbiome dysbiosis is linked to conditions including Alzheimer’s disease, Parkinson’s disease, depression, and anxiety, possibly via immunological, inflammatory, and neurological mechanisms (97). Oral dysbiosis has been associated with pregnancy-related difficulties such as preterm birth, low birth weight, and gestational inflammatory disorders, potentially via the hematogenous dissemination of bacteria and inflammatory mediators to placental tissues (98, 99).

**Figure 3:**
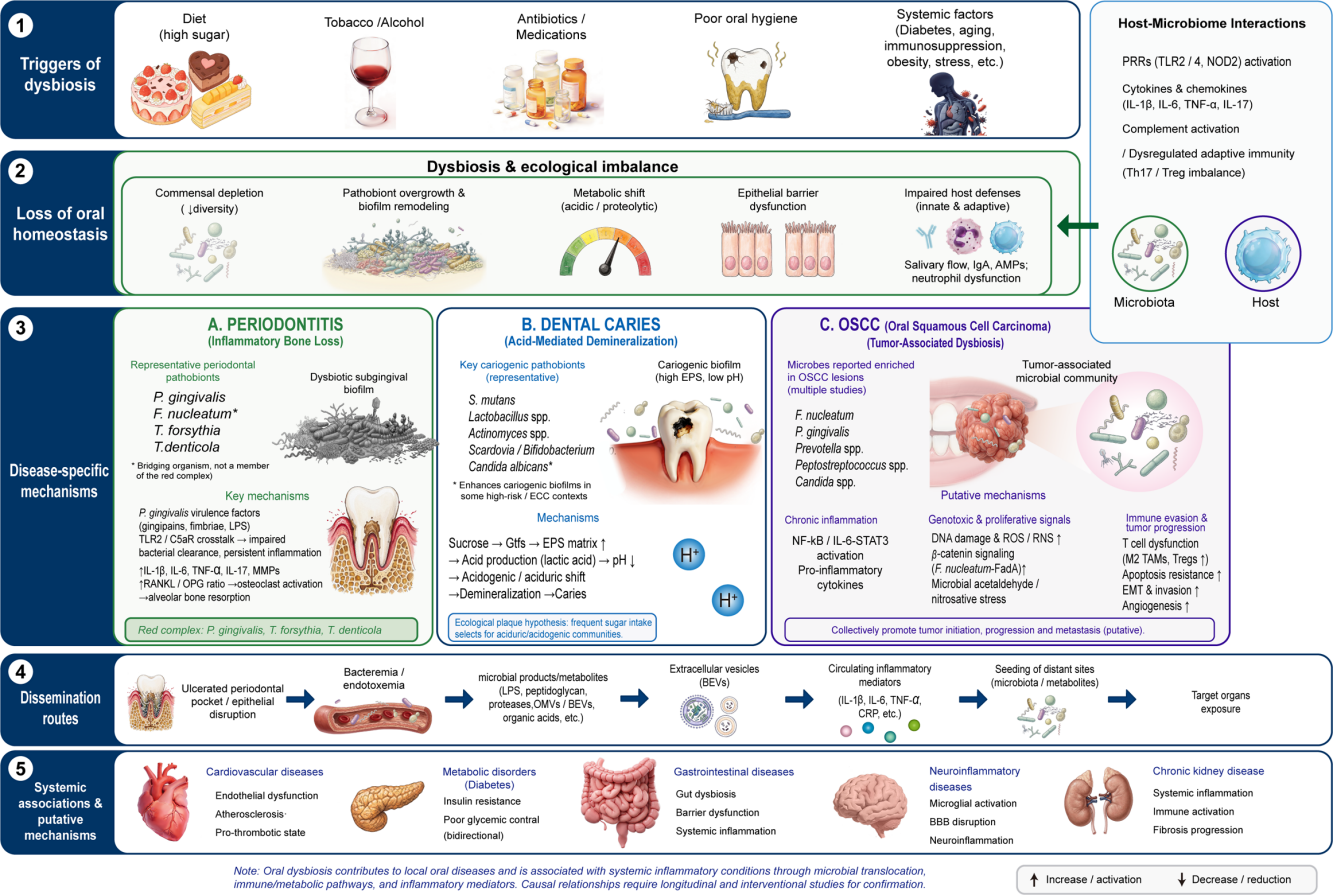
Oral microbiome dysbiosis in disease pathogenesis and systemic inflammation. Oral microbiome dysbiosis can be initiated by dietary, behavioral, iatrogenic, hygiene-related, and systemic factors, including high-sugar intake, tobacco or alcohol exposure, antibiotics or medications, poor oral hygiene, diabetes, aging, immunosuppression, obesity, and stress. These triggers disrupt oral ecological balance through commensal depletion, pathobiont expansion, biofilm remodeling, metabolic acidification or proteolytic shifts, epithelial barrier dysfunction, and impaired innate or adaptive host defenses. Dysbiotic microbial communities interact with host immune pathways through PRR activation, inflammatory cytokine and chemokine production, complement activation, and Th17/Treg imbalance. These processes contribute to disease-specific mechanisms in periodontitis, dental caries, and OSCC, including inflammatory bone loss, acid-mediated demineralization, tumor-associated microbial remodeling, chronic inflammation, genotoxic stress, and immune evasion. Local dysbiosis may further extend beyond the oral cavity through bacteremia, endotoxemia, microbial products or metabolites, extracellular vesicles, and circulating inflammatory mediators, thereby contributing to systemic inflammatory associations involving cardiovascular, metabolic, gastrointestinal, neuroinflammatory, and renal diseases. Causal relationships require further validation in longitudinal and interventional studies.

These findings indicate that the mouth cavity serves as both a localized microbial habitat and a clinically significant interface between mucosal ecology and systemic inflammatory health, affecting several systems (**Figure 3**). **Table 2** gives an organized summary of the most important oral microbes, the diseases they linked to, how they contribute to these diseases, and how they spread. This table highlights how some commensal microorganisms can become pathogenic under certain conditions, emphasizing the importance of maintaining a balanced oral microbiome to prevent both oral and systemic diseases.

**Table 2:** Impact of oral microbial imbalance on disease: key pathogens and mechanisms.

Microbial taxon	Associated diseases	Key pathogenic mechanisms	Transmission routes	Reference
Porphyromonas spp.	Chronic periodontitis, aggressive periodontitis	Secretion of gingipains (proteases) degrading collagen; LPS-induced inflammatory activation leading to periodontal tissue destruction	Direct contact (saliva, plaque, GCF); vertical transmission (mother-child, family); sexual contact	(100)
Fusobacterium spp.	Periodontitis, pericoronitis, oral and maxillofacial infections	Acts as a “bridging organism” linking early and late colonizers; adhesion factors promote biofilm formation and enhance pathogenicity	Saliva, plaque, direct contact; vertical transmission; oral-gut axis transmission	(101)
Treponema spp.	Periodontitis, Acute Necrotizing Ulcerative Gingivitis (ANUG)	Spirochete motility Protease secretion (Dentilisin) Immune modulation	Direct contact; Vertical transmission; Iatrogenic transmission (dental instruments)	(102)
Tannerella forsythia	Chronic periodontitis, periodontal abscess	Fibrinogen-binding proteins; virulence factors Leucine-rich repeat protein (BspA)	Direct contact; horizontal transmission within families	(103)
Streptococcus mutans	Dental caries	Production of extracellular polysaccharides for biofilm formation; lactic acid production lowers pH (<5.5), causing enamel demineralization	Direct contact (saliva, plaque); vertical transmission (mother-child); indirect transmission via food/utensils	(104)
Candida spp. (fungi)	Oral candidiasis (thrush), denture stomatitis	Hyphal invasion of epithelium; secretion of aspartyl proteases disrupting mucosal barrier; Morphological transition; Production of virulence factors	Vertical transmission (birth, breastfeeding); direct contact; iatrogenic; endogenous dysbiosis	(105)
Prevotella spp.	Periodontitis, Gingivitis	Through enzyme-mediated degradation of host tissues; causing dysbiosis of the oral microbiome and triggering inflammatory responses	Direct contact (e.g., saliva transmission), vertical transmission	(106)
Aggregatibacter actinomycetemcomitans	Aggressive periodontitis	Leukotoxin induces immune cell apoptosis and tissue destruction	Vertical transmission (saliva, kissing); close contact; sexual transmission	(106)
Lactobacillus spp.	Deep caries, root caries, pulpitis	Strong acid tolerance (pH ~3.8); continuous lactic acid production; proteolytic degradation of dentin matrix	Generally non-transmissible (commensal); dietary intake; vertical transmission	(107)
Campylobacter spp.	Chronic periodontitis, gingivitis	LPS activates TLR4 pathway; hydrogen peroxide damages epithelial cells	Direct contact; fecal-oral route; contaminated food/water	(108, 109)
Actinomyces spp.	Root caries, gingivitis	Adhesive polysaccharide production; weak but persistent acid production causing demineralization	Early colonizer; vertical transmission; endogenous infection	(110, 111)
Haemophilus spp.	Peri-implant mucositis, gingivitis	Adhesins facilitate colonization; metabolic byproducts induce epithelial inflammation	Direct contact; droplets; close contact (kissing, utensils)	(112, 113)
Staphylococcus spp.	Oral mucosal abscess, periodontal abscess	α-toxin damages cells; coagulase promotes immune evasion	Direct contact; contaminated surfaces; iatrogenic; rare airborne transmission	(106, 114)
Klebsiella spp.	Postoperative oral infection, maxillofacial space infection	ESBL-mediated antibiotic resistance; capsule resists phagocytosis	Iatrogenic; environmental sources; fecal-oral route; direct contact	(115)
Pseudomonas spp.	Oral mucosal infection, peri-implantitis (immunocompromised)	Pyocyanin and elastase-mediated tissue damage; biofilm formation increases resistance	Iatrogenic (devices, water systems); environmental exposure; opportunistic infection	(116)
Peptostreptococcus spp.	Periodontal abscess, osteomyelitis	Organic acid production lowers pH; synergistic pathogenicity promotes abscess formation	Direct contact; iatrogenic; endogenous infection (gut translocation)	(117)

In a healthy oral microbiome, various microorganisms collectively maintain ecological balance and contribute beneficial functions. However, under the influence of specific environmental or external factors, alterations in the oral environment can lead to the transformation of initially harmless commensals into pathogenic microbes. This shift occurs through ecological dysbiosis and changes in immune responses, which promote localized inflammatory reactions, subsequently leading to oral diseases. Therefore, the transition from a balanced to a pathogenic state in microorganisms is primarily driven by a complex interplay between host conditions and external environmental factors.

## 5 Therapeutic recalibration of the oral ecosystem

Therapeutic strategies targeting oral dysbiosis are increasingly being reframed within a systems-level perspective, in which oral diseases are viewed as disorders of host-microbiome-immune disequilibrium rather than simple infections. Accordingly, effective treatment should not only reduce pathogenic burden but also restore ecological balance and recalibrate host responses. In this context, current and emerging therapies can be broadly organized into ecological restoration, pathogen-selective suppression, host-inflammatory modulation, and immune regulation.

Conventional approaches such as mechanical debridement and antimicrobial therapy mainly reduce microbial load, whereas newer strategies, including probiotics, prebiotics, host-modulatory therapies, pro-resolving mediators, and microbiome engineering, aim to restore host-microbiota compatibility. These approaches are best viewed as complementary rather than mutually exclusive.

### 5.1 Ecological restoration strategies

#### 5.1.1 Probiotics, prebiotics, and synbiotics

Beneficial commensals may compete with pathogens, produce antimicrobial compounds, and modulate host immunity. Probiotic strains (e.g. *Lactobacillus reuteri*, *L. casei*, *L. rhamnosus*, *Bifidobacterium* spp., *Streptococcus salivarius* K12) have been tested as adjuncts in periodontal therapy. These organisms can colonize the biofilm and secrete bacteriocins (e.g. reuterin from *L. reuteri*) that inhibit pathogens (118). Clinically, probiotic supplementation (often as lozenges or gels) has produced modest but significant improvements in gingival inflammation and bleeding scores, and reduced levels of periodontal pathogens. For example, meta-analyses report that adjunctive *Lactobacillus* and *Bifidobacterium* probiotics lower gingival bleeding and decrease *Porphyromonas gingivalis* counts in plaque (119, 120). Adjunctive probiotics may confer modest benefits, but strain-specific conclusions remain limited by study heterogeneity.

Prebiotics, like arginine, are substances that don’t break down in the body and help beneficial commensals grow. Bacteria in the mouth break down arginine using the arginine deiminase (ADI) pathway, which makes ammonia. This increases local pH and suppresses the expansion of acidogenic taxa. Arginine also stops *P. gingivalis* from clumping together with other anaerobes, perhaps by messing with gingipains. ADI from *Streptococcus intermedius* stops *P. gingivalis* biofilm from growing by turning down the fimA and mfa1 genes, which are needed for making fimbriae. This impact relies on the enzymatic activity of ADI; a mutant variety of ADI (ADIC399S) is incapable of catalyzing arginine hydrolysis, hence failing to impede biofilm formation. Biofilm formation does not occur in arginine-depleted environments but can be reinstated with arginine, underscoring its function as a signal for P. gingivalis biofilm development. These results suggest that ADI affects biofilm formation by lowering arginine levels, offering new understanding of how bacteria communicate in oral diseases linked to biofilms (121).

Oral disorder like tooth decay and gum disease are increasingly linked to an imbalance in the mouth’s bacteria, where this imbalance disrupts how microbe function and affects the body’s immune system. Nitrate has been found to alter the environment of oral bacteria without affecting the overall growth of biofilms. It reduces lactate levels, while increasing ammonium levels and raising the pH. It selectively boosts health-associated nitrate-reducing genera like *Neisseria* and *Rothia* while suppressing taxa linked to caries and periodontitis, which supports its possible role as a prebiotic for oral health (25).

Probiotics and other microbial treatments fight infections and modulate the immune system, which is helpful for tooth health. Studies have shown that taking probiotics can boost mucosal immunity by increasing sIgA levels in saliva. This is important for neutralizing harmful microbes on the surface of mucous membranes and maintaining a healthy balance of bacteria. For example, both live and heat-treated probiotics can increase Immunoglobulin A (IgA) levels in saliva and trigger the release of immune-regulating molecules like Interleukin-10 (IL-10). This means they might help reduce inflammation and improve immune balance in the mouth (122, 123). These immune-mediated pathways enhance the microbial ecological effects of probiotics and may assist in alleviating dysbiosis-associated inflammation in the oral cavity.

#### 5.1.2 Oral microbiome transplantation

Microbiome transplantation (MT) aims to restore the natural balance of bacteria, improving immune function and helping treat illnesses caused by microbial imbalances (124). In OMT, healthy, diverse microbes from a donor are transferred into the mouth of someone with oral issues. This helps restore the microbial balance, addressing problems like gum disease and tooth decay, which are associated with disrupted bacterial balance leading to inflammation, tissue damage, and disease progression (125).

A published study protocol has proposed samples of supragingival plaque were taken from 600 healthy people. We will sequence these samples to find out what makes up a healthy microbiome. The samples will then be cultured in an *in vitro* flow-cell model for 14 days to expand the microbial community and confirm microbiota viability. The healthiest bacteria will be put into a hydrogel delivery device for transplanting. Lastly, animal models of caries and periodontitis will be used to test how well and safely OMT works (126). Therefore, although OMT provides a promising conceptual strategy for restoring oral microbial balance, it remains at an early investigational stage, and further studies are needed to establish its safety, ecological stability, delivery methods, and clinical efficacy in humans.

#### 5.1.3 Biofilm-disrupting peptides and enzymes

Oral chronic infections are strongly linked to the growth of dental plaque biofilms, which make bacteria more resistant to both antimicrobial treatments and the body’s defenses. Antibiotic overuse has exacerbated this challenge by promoting antimicrobial resistance (AMR), thereby complicating the treatment of biofilm-associated oral diseases. AMPs have been popular as possible medicines since they work against a wide range of diseases, are unlikely to cause resistance, and are safe for the body (127).

For instance, enzymes such as DNases and dispersin B degrade the biofilm matrix, thereby increasing microbial susceptibility to antimicrobial clearance. Dispersin B and DNase are enzymes that are very important for breaking up bacterial biofilms. Dispersin B selectively breaks down poly-N-acetylglucosamine (PNAG), which is an important part of the biofilm matrix. This lets bacterial cells spread out, leaving them more vulnerable to antimicrobial drugs. Similarly, DNase degrades extracellular DNA, another critical element in biofilm structure, weakening the biofilm and enhancing the effectiveness of antibiotics. Both enzymes show promise in improving the treatment of biofilm-related infections by making bacteria more vulnerable to antimicrobial therapies (128).

All these precision approaches are largely experimental, but they hold promise. By specifically eradicating pathogens or restoring healthy commensals, they aim to shift the oral ecosystem back to a disease-resistant state without broadly wiping out the microbiota.

### 5.2 Pathogen-selective suppression

#### 5.2.1 Bacteriophage therapy and antimicrobial photodynamic therapy

Bacteriophage therapy, as an innovative treatment for PD, holds great potential, especially in cases of AMR. Bacteriophages have shown significant efficacy in inhibiting the growth of periodontal pathogens such as *Aggregatibacter actinomycetemcomitans*, *Fusobacterium nucleatum*, *Streptococcus gordonii*, and *Porphyromonas gingivalis*. 8 studies met the inclusion criteria, employing 11 different bacteriophages, with the primary evaluation methods being optical density and colony-forming unit (CFU) measurements. While these results demonstrate efficacy preclinical *in vitro*, further research is needed to validate the effectiveness of bacteriophage therapy in multispecies biofilms and clinical settings (129). Oral infections are frequent, and as antibiotic resistance grows, more people are looking into other treatments like aPDT. In gum disease, aPDT helps get rid of pathobionts and supports tissue healing. It uses light and photosensitizers. This study examines the determinants influencing aPDT efficacy, photosensitizers, and recent innovations, emphasizing novel clinical applications and the synergistic effects of aPDT with chemical and biomolecular agents (130).

#### 5.2.2 Clustered regularly interspaced short palindromic repeats-CRISPR-associated proteins antimicrobials and engineered microbiome therapeutics

The rise of antibiotic-resistant bacteria is a global public health threat, and the slow progress in discovering new antibiotics calls for alternative strategies. One promising approach is the use of CRISPR-Cas based systems for precisely targeting and eliminating bacterial populations. This system’s versatility and specificity allow for selective inactivation of genes involved in antibiotic resistance, biofilm formation, virulence, and bacterial survival. CRISPR-Cas can work through two main mechanisms: by disrupting chromosomal genes or by removing antibiotic resistance plasmids (131). Beneficial bacteria can be genetically modified to enhance their therapeutic action. For instance, oral *Streptococcus* strains are being engineered to **secrete high levels of bacteriocins** or even CRISPR antimicrobials against competitors. Innovative strategies, such as engineered probiotics and CRISPR-based microbiome engineering, show promise as next-generation therapeutic approaches. Despite these advancements, the translation of microbiome-based therapies into clinical applications still faces challenges due to ethical, regulatory, and ecological barriers (132).

In particular, CRISPR-Cas systems have shown great potential in combating AMR by targeting and eliminating antibiotic-resistant genes, such as the successful targeting of colistin resistance genes on MCR-1 plasmids. However, their effectiveness can be influenced by variations in CRISPR loci across different bacterial species. Despite these promising results, challenges persist, such as optimizing delivery methods and minimizing off-target effects, to ensure the safety and precision of CRISPR-Cas systems for clinical use (133).

### 5.3 Host-directed inflammatory recalibration

#### 5.3.1 Nonsteroidal anti-inflammatory drugs

NSAIDs reduce inflammation and pain by inhibiting the activity of cyclooxygenase (COX−1 and COX−2), thereby reducing the production of inflammatory mediators, particularly prostaglandins, a mechanism that has been widely confirmed. COX−2 is highly expressed at inflammatory sites, and the prostaglandin E2 (PGE2) it produces plays a critical role in periodontal inflammation and bone resorption. By blocking this pathway, NSAIDs can alleviate gingival inflammation and delay pathological bone loss (134). Clinical and preclinical studies show that, as adjunctive therapy in routine periodontal treatment, NSAIDs (such as aspirin, ibuprofen, naproxen, diclofenac, and selective COX−2 inhibitors) can improve gingival inflammation markers, reduce probing depth bleeding, and, in some small-scale trials, show protective effects against alveolar bone loss. Long-term or periodic administration may even significantly improve clinical parameters in certain cases. On the other hand, as NSAIDs also inhibit the production of physiological prostaglandins, their long-term use increases gastrointestinal side effects and cardiovascular risks, limiting their widespread application in chronic PD management. Research on localized delivery strategies (such as gels or mouthwashes) and optimizing dosages for different patient groups remains a future direction for exploring safer and more effective use of NSAIDs in periodontal treatment (135).

#### 5.3.2 Doxycycline-based host-modulatory therapy

A small randomized, double-blind, placebo-controlled study evaluated the effect of Low-dose doxycycline (LDD) in combination with non-surgical periodontal therapy for patients with chronic periodontitis. 30 patients were randomly assigned to the LDD group and the placebo group. The LDD group took 20 mg of doxycycline every day, along with a cleaning procedure called scaling and root planing (SRP). The findings indicated that, over a 12-month duration, the LDD group exhibited a significant enhancement in probing depth and gingival index relative to the placebo group, while Matrix Metalloproteinase-8 (MMP-8) levels in gingival crevicular fluid (GCF) were markedly reduced in the LDD group at 6 months. This means that LDD can be a useful addition to periodontal therapy, as it can lower MMP-8 levels and improve clinical indicators. This is good evidence for the long-term management of PD (136). This evidence underscores the possibility of low-dose LDD as an additional therapy for periodontitis. Sub-antimicrobial dosage doxycycline (SDD), which also lowers matrix metalloproteinases (MMPs) that cause tissue destruction in periodontitis, has also demonstrated good outcomes. Research indicates that the combination of SDD (20 mg twice daily) with SRP results in substantial enhancements in clinical attachment and probing depths. It is important to note that SDD does not have any antibacterial properties, does not cause resistance, and has very little negative effects (137).

#### 5.3.3 Specialized pro-resolving lipid mediators

Molecules such as resolvins and lipoxins actively facilitate the resolution of inflammation. Resolvin E1 (RvE1) therapy significantly inhibited and restored bone loss induced by periodontitis in preclinical mammalian models. Local therapy with RvE1 substantially decreased the expression of inflammation-associated genes, reverting the gene expression profile to a state more akin to health. Additionally, RvE1 treatment significantly decreased the density of osteoclasts and reduced the infiltration of inflammatory cells. After treatment, the rat subgingival microbiota underwent significant changes, indicating that modulation of local inflammation plays an important role in shaping the composition of the subgingival microbiota. These findings support the potential of RvE1 in modulating inflammation and improving periodontal health (138). Further research has demonstrated that RvE1, a proresolving mediator derived from omega-3 eicosapentaenoic acid, also shows promise in treating established periodontitis in rabbits. In contrast to pro-inflammatory lipids such as PGE (2) and leukotriene B (4), which exacerbate periodontitis, RvE1 promotes the resolution of inflammation, leading to complete restoration of the local lesion and a reduction in systemic inflammatory markers like CRP and IL-1beta. This is the first report showing that RvE1, as a naturally occurring lipid mediator, can regenerate pathologically lost tissues, including bone, by resolving inflammation (139).

#### 5.3.4 Metabolic and bone-active host modulators

Vitamin D plays a key role in periodontal health due to its anti-inflammatory and host-modulatory effects. Studies have shown that vitamin D deficiency is linked to more severe PD, and its supplementation can improve healing outcomes. Vitamin D supplementation (VDS) has been found to regulate immune responses, inhibit pro-inflammatory cytokines, and decrease levels of inflammatory markers like IL-1, IL-6, (TNF-α), and IL-10 in periodontitis patients. VDS also boosts antimicrobial action by encouraging the release of AMPs and proteins involved to autophagy, which help combat pathobionts like *Aggregatibacter actinomycetemcomitans* and *Porphyromonas gingivalis.* Clinical research indicated that VDS enhances clinical results, including diminished clinical connection loss and probing depth. These findings highlight the potential of vitamin D as an adjunct therapy for PD, offering benefits through its anti-inflammatory, host-modulatory, and antimicrobial actions (140).

Statins modulate inflammation, immune response, bone metabolism, and bacterial clearance through multiple pathways. They control periodontal inflammation by inhibiting pro-inflammatory cytokines and promoting the release of anti-inflammatory and pro-resolution molecules, mainly through the Extracellular Signal-Regulated Kinase (ERK), Mitogen-Activated Protein Kinase (MAPK), Phosphoinositide 3-Kinase-Protein Kinase B (PI3-Akt), and Nuclear Factor Kappa-B (NF-κB) pathways. Additionally, they regulate the host response activated by bacterial challenges, preventing inflammation-mediated bone resorption and promoting bone formation. Furthermore, they reduce bacterial growth, disrupt bacterial membrane stability, and enhance bacterial clearance, thereby preventing the exacerbation of infection. Compared to systemic delivery, local statin application as an adjunct to periodontal therapy results in better treatment outcomes. Moreover, combining statin therapy with other regenerative agents improves the periodontal healing response. Therefore, statins could be proposed as a potential adjuvant to periodontal therapy. However, optimizing the combination of their dose, type, and carrier could be crucial in achieving the best treatment response (141).

#### 5.3.5 Cytokine- and bone-targeted emerging therapies

Antagonists of TNF−α and IL−1β or their receptors can attenuate inflammatory responses and may limit periodontal tissue destruction. However, most of these approaches remain at the preclinical or experimental stage, and further randomized controlled trials are needed to confirm their safety and effectiveness in clinical periodontitis treatment (142).

Natural bioactive compounds and specialized pro−resolving mediators have emerged as promising adjunctive treatments for PD. Pro-resolving mediators generated from omega-3 fatty acids, including resolvins, protectins, and maresins, actively facilitate the resolution of inflammation and maintain tissue homeostasis in experimental models (143).

Therapeutic methods that obstruct the RANKL pathway, including anti-RANKL antibodies, impede osteoclastogenesis and mitigate inflammation-induced bone resorption. Denosumab, a completely human monoclonal antibody against RANKL, binds to RANKL and stops it from interacting with RANK. This lowers osteoclastogenesis and bone resorption (144). In preclinical models, denosumab has been demonstrated to lower the activity of osteoclasts and bone turnover, raise bone mass, and enhance bone microarchitecture. It is clinically approved for treating osteoporosis and other osteolytic diseases (145).

These therapeutic strategies, including cytokine-targeting biologics and bioactive small molecules, represent promising adjunctive approaches for PD by modulating inflammation, immune responses, and bone metabolism; nevertheless, additional study is required to enhance their clinical use.

### 5.4 Immunotherapy and immune-reprogramming strategies

#### 5.4.1 Prophylactic and therapeutic vaccination strategies

Vaccination against significant oral pathogens is being investigated as a prophylactic approach for disorders associated with oral biofilms. In periodontitis, *Porphyromonas gingivalis* has been a principal vaccination target. Experimental investigations have demonstrated that antigens such as capsular polysaccharide, fimbriae, and gingipains can elicit protective immune responses and mitigate alveolar bone loss in animal models. In the case of dental caries, most of the work on vaccines has been done on *Streptococcus mutans*. Studies have demonstrated that inducing immunity against specific surface proteins or enzymes can elevate IgA levels in saliva, diminish bacterial accumulation, and mitigate the severity of dental caries in animals (146). Vaccines aimed at *Streptococcus mutans* surface proteins have demonstrated potential in experimental models of dental caries. By inducing salivary IgA and serum IgG responses against key adhesion-related antigens, such vaccines may reduce bacterial colonization and cariogenic lesion development, supporting their potential as preventive strategies against caries (147).

Dental caries, one of the most common bacterial infections worldwide, has been a major focus of vaccine research, particularly targeting *Streptococcus mutans*, a key cariogenic pathogen. Preclinical studies and early clinical evidence suggest that mucosal immune responses against important S. mutans antigens may help interfere with bacterial colonization and cariogenic processes. Although no vaccine has yet reached routine clinical use, these findings support the potential of prophylactic immunization as a strategy to prevent oral dysbiosis and biofilm-related oral diseases (148).

#### 5.4.2 Immune checkpoint and cancer immunotherapy

In OSCC, immunotherapy has become an important treatment option, especially for patients with recurrent, metastatic, or unresectable disease. Immune checkpoint inhibitors (ICIs), particularly the anti- Programmed Cell Death Protein 1 (PD-1) antibodies pembrolizumab and nivolumab, have shown survival benefits in recurrent/metastatic head and neck squamous cell carcinoma (HNSCC), including OSCC as a major subtype, and are now integrated into standard treatment strategies (149). These agents work by blocking inhibitory immune signaling, thereby restoring T-cell-mediated anti-tumor activity and enhancing immune recognition of tumor cells. KEYNOTE-048 established pembrolizumab, alone or with chemotherapy, as a first-line standard of care for recurrent or metastatic HNSCC, particularly in patients with Programmed Death-Ligand 1 (PD-L1)-positive tumors (150). Importantly, long-term follow-up from KEYNOTE-048 further confirmed that pembrolizumab-based first-line therapy provided durable survival benefit in recurrent/metastatic HNSCC, while patients also maintained favorable outcomes with subsequent lines of treatment (151). CheckMate 141 established nivolumab as an effective treatment for platinum-refractory recurrent or metastatic HNSCC by improving survival with lower toxicity (152). Although clinical benefit of ICIs is well-supported in HNSCC/OSCC populations, these trials did not stratify patients by microbiome status.

Recent studies have highlighted that oral microbial dysbiosis may contribute to cancer progression and immune remodeling, supporting the concept that the oral microbiome could serve as both a biomarker and a therapeutic target in cancer, including in the context of immunotherapy (153). A small tissue-based microbiome study comparing 10 paired OSCC tumor and adjacent non-tumor mucosal samples (collected 5 cm from the tumor margin) identified clear differences in bacterial composition between the two sites. Using 16S rRNA-based analysis, the authors identified 1,200 sequences and discovered 80 bacterial species/phylotypes across 6 phyla, including *Firmicutes*, *Bacteroidetes*, *Proteobacteria*, *Fusobacteria*, and *Actinobacteria*. Several species were identified as abundant in tumor tissues, while *Granulicatella adiacens* was predominantly detected in non-tumor mucosa. *Streptococcus intermedius* was found in 70% of both tumor and non-tumor tissues. These findings suggest that OSCC is linked to a change in the types of bacteria in the mouth, supporting the idea that microbial imbalance could play a role in the development of oral cancer (154).

#### 5.4.3 Immune tolerance and regulatory therapies

Therapies that enhance host regulatory responses may help reduce chronic inflammation and restore periodontal immune homeostasis. Because immune tolerance is essential for maintaining host-microbiota balance in the oral cavity, Regulatory T cells (Tregs) have emerged as a promising target for controlling chronic oral inflammatory diseases.

For example, in a ligature-induced murine model of periodontitis, local delivery of Treg-inducing factors significantly reduced inflammation and prevented alveolar bone loss, supporting immune regulation as a promising therapeutic strategy; notably, this effect was observed in a preventive, experimental setting rather than in established disease (155). This “tolerance-based” approach aims not simply to eliminate bacteria, but to rebalance host-microbiota interactions and reduce destructive inflammation (156). In the future, microbial adjuvants, immunomodulatory peptides, or other mucosal tolerance-inducing strategies may offer additional ways to promote peaceful coexistence with the normal oral flora and improve long-term periodontal stability.

#### 5.4.4 Microbiome-immune interactions and emerging biologicals

Studies of human gingival tissues and monocytes suggest that the oral mucosa may develop a form of endotoxin tolerance during sustained exposure to bacterial components such as LPS, thereby downregulating TLR signaling and inflammatory cytokine responses to help limit excessive local inflammation (157).

Recent studies suggest that resident microbiota can shape mucosal immune homeostasis by regulating key immune cell populations such as Tregs and Th17 cells. Commensal microorganisms may affect the balance of inflammation, immunological tolerance, and tissue resilience through direct interactions with the host and bioactive chemicals produced by microbes. This shows that microbiota-based immune regulation could be a useful treatment (158).

Together, these findings suggest that restoring mucosal immune tolerance and host-microbiota balance may represent a promising strategy for controlling periodontal inflammation. By harnessing the immune system-either through vaccines, checkpoint modulators, or regulatory cell expansion. It could be able to teach the host to coexist peacefully with commensals while effectively eliminating infections.

### 5.5 Precision/next-generation interventions

The integration of multi-omics and AI is increasingly enabling personalized approaches to oral disease prevention, diagnosis, and treatment. Multi-omics helps us closely study a person’s biological traits, disease risks, and oral bacteria, providing important markers and tools to predict and understand complex oral problems. By merging advanced imaging data (such as Cone Beam Computed Tomography (CBCT) and Magnetic Resonance Imaging (MRI)) with AI-driven data analysis platforms, the genetic information, clinical features, imaging characteristics, and molecular omics data of patients can be integrated into high-dimensional datasets. Learning-based models can identify diagnostic and therapeutic patterns, supporting disease classification, treatment-response prediction, and individualized management of oral diseases. This method makes things more accurate and opens up the prospect of tailored treatment programs that can change over time. AI-based risk prediction models and multi-omics feature analyses also help make changes to clinical therapies on the spot. This could revolutionize how we treat people from a standard way to a personalized oral health care system that is “tailored” to each person (159).

AI-assisted CBCT has been applied to dental implant planning by automatically assessing bone height, bone thickness, missing tooth regions, and adjacent anatomical structures. In a study of 75 CBCT scans and 508 implant sites, the AI system accurately detected missing tooth regions in 95.3% of cases and showed comparable bone height measurements in several regions (160). In a deep learning study of Temporomandibular Joint (TMJ) MRI scans from two hospitals, AI models automatically identified regions of interest, segmented key joint structures, and classified anterior disc displacement with precision rates above 92%. This suggests that AI-MRI may support more standardized and accurate diagnosis of TMJ disorders in clinical practice (161).

Rather than relying solely on broad-spectrum antimicrobials, emerging interventions aim to selectively target microbial ecology and host immune responses. We now aim to restore the natural balance of germs, boost the mouth’s defense, and improve the body-bacteria interaction rather than eradicate unwanted bacteria. **Table 3** highlights the main treatment methods commonly used in clinics to address oral microbial imbalances and how they can be helpful.

**Table 3:** Representative strategies of key clinical interventions for oral dysbiosis.

Therapeutic approach	Diseases/indications	Specific treatment interventions	Main clinical/biological effects	Clinical stage/status	Representative references
Mechanical Debridement (SRP)	Periodontitis	SRP, subgingival debridement	Reduces pathogen load, improves inflammation markers	Standard clinical treatment	(162)
Antimicrobial Systemic/Topical Therapy	Periodontitis, Aggressive Periodontitis	Local chlorhexidine, amoxicillin, metronidazole, etc.	Reduces pathogenic bacteria	Common clinical use (with risk of resistance)	(163)
Probiotics	Periodontitis, Gingivitis, Dental Caries	Probiotic strains such as Lactobacillus spp., Streptococcus salivarius	Inhibits pathogenic bacteria, improves inflammation, restores microbiota balance	Mostly clinical trials/adjunct therapy	(164)
Prebiotics	Oral microbiome Imbalance	Oligosaccharides (FOS, GOS), amino acid derivatives	Promotes growth of beneficial bacteria, improves ecological balance	Experimental/early clinical	(165, 166)
Synbiotics	PD/Caries	Probiotics + Prebiotics	Synergistically enhances probiotic colonization and function	Clinical/experimental combination studies	(167, 168)
Postbiotics	Periodontitis	Bacterial metabolites/dead bacteria components	Regulates immunity, inhibits pathogen proliferation	Clinical adjunct/research phase	(169–171)
AMPs/Compounds	Dental Caries, Periodontitis	AMPs, specific antimicrobial peptide formulations	Selectively inhibits pathogens, disrupts biofilm formation	Experimental/early clinical	(127, 172)
Nanodrug Delivery Systems	Periodontitis, Peri-implantitis	Nanocarrier hydrogels, responsive hydrogels	Targeted drug release, promotes tissue regeneration	Animal/early clinical	(173, 174)
OMT	Imbalanced Periodontitis/Caries	Transplantation of healthy microbiota	Rebuilds ecological structure, rejects pathogens	Preliminary research/future direction	(125, 175, 176)
Phage Therapy	Pathogen-specific Pathogens	Phages targeting P. gingivalis, etc.	Precisely eliminates specific pathogens	Preclinical/experimental phase	(129, 177)
Host Inflammation Modulation (SPMs)	Chronic Periodontitis	Resolvin, Maresin, etc.	Promotes inflammation resolution, tissue repair	Experimental/early clinical	(178)
Immune Checkpoint Modulation	Peri-implantitis/Periodontitis	Anti-PD-1/modulatory immune strategies	Modulates immune response, slows lesion progression	Research phase	(179, 180)
Laser Treatment	Periodontitis/Peri-implantitis	Laser-assisted lesion removal	Reduces bacterial load, enhances healing	Clinical adjunct	(181)
Oral Rinses	Gingivitis/Plague Control	Chlorhexidine, essential oils, etc. mouthwash	Reduces plaque, short-term antimicrobial effect	Widely used	(182, 183)
Fluoride/Salivary Regulators	Dental Caries	Fluoride gels/mouth rinses	Enhances acid tolerance, reduces demineralization	Routine prevention/clinical	(184)
Lifestyle Modifications	All Oral Diseases	Diet, oral hygiene, smoking cessation	Improves microbiota stability and immune barrier	Public health/clinical	(185, 186)
Local Immune Modulation	Oral Inflammation	TLR antagonists, etc.	Reduces excessive host inflammatory response	Experimental phase	(187, 188)
Biofilm Disruptors	Biofilm-related Diseases	Enzyme preparations, nano EPM disruptors	Disrupts pathogenic biofilm structure	Experimental phase	(189, 190)
Modified Implant Surface Materials	Peri-implantitis	Antimicrobial/osteointegration-promoting material coatings	Reduces bacterial adhesion, enhances bone integration	Development/clinical pilot	(191)
Oral Vaccine Strategies (Candidates)	Specific Pathogen Infections	Vaccines targeting P. gingivalis, S. mutans, etc.	Induces protective immunity, reduces bacterial colonization	Preclinical	(148)
Biofilm-Disrupting Peptides	Chronic Oral Infections	Dispersin B, DNase	Disrupts biofilm matrix, enhances microbial clearance	Experimental/early clinical	(128)
CRISPR-Cas Antimicrobials	Antibiotic-resistant bacteria	CRISPR-Cas-based systems	Selectively inactivates antibiotic resistance and biofilm formation genes	Experimental	(131–133)
NSAIDs	Chronic Periodontitis	Topical delivery of NSAIDs	Reduces inflammation and bone resorption	Development/Clinical adjunct	(134, 135)
Doxycycline-Based Host-Modulatory Therapy	Chronic Periodontitis	LDD	Reduces MMP-8 levels, improves clinical parameters	Clinical trials	(136, 137)
Pro-Resolving Lipid Mediators	Periodontitis	Resolvin, RvE1	Promotes inflammation resolution, tissue repair	Experimental/early clinical	(138, 139)
Cytokine- and Bone-Targeted Emerging Therapies	Periodontitis, Osteoporosis	TNF-α, IL-1β antagonists, anti-RANKL antibodies	Reduces inflammation, prevents bone resorption	Preclinical/experimental	(144, 145)

**Figure 4:**
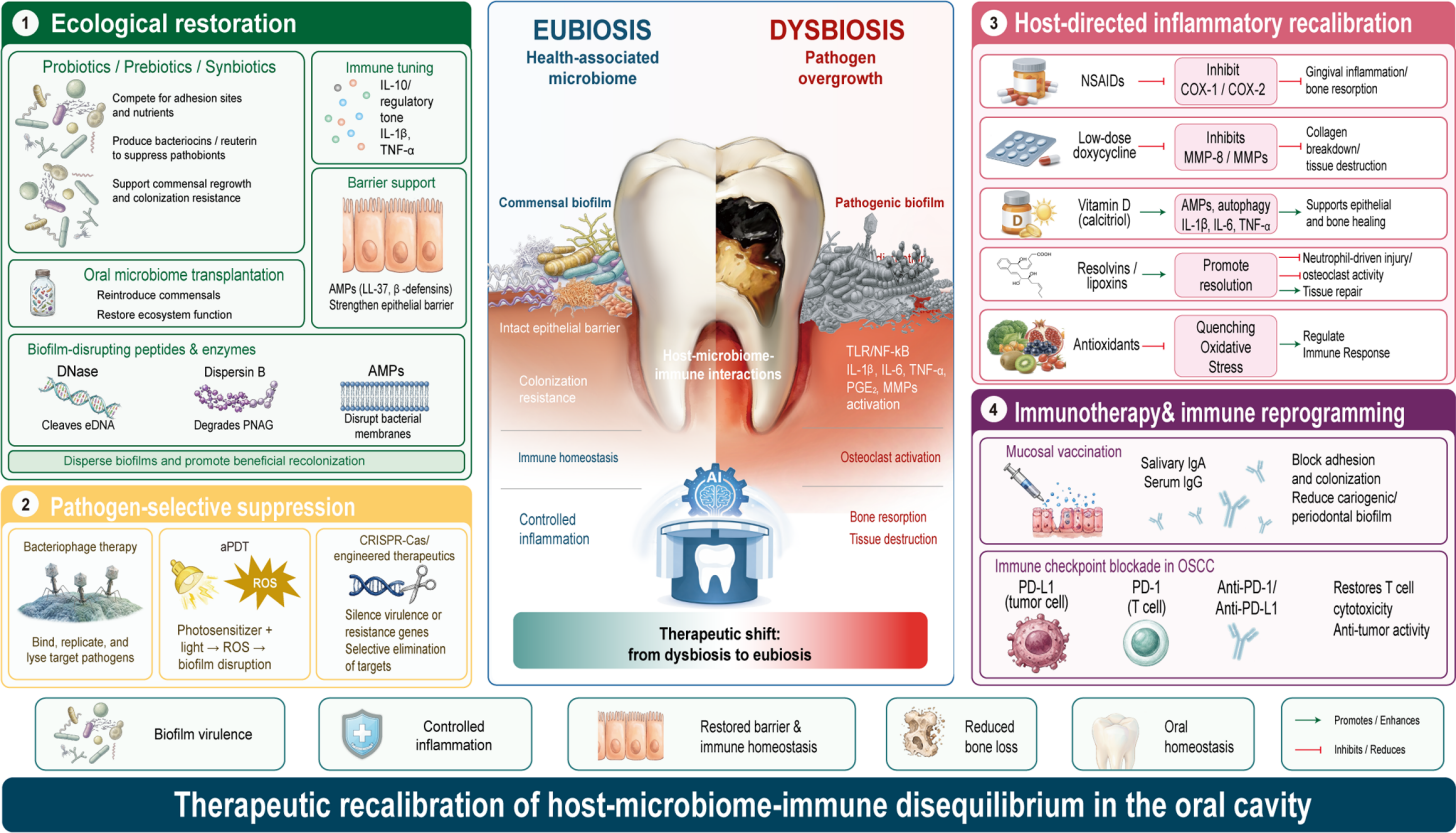
Therapeutic strategies targeting host-microbiome-immune disequilibrium in oral diseases. Therapeutic strategies for oral dysbiosis aim to restore health-associated eubiosis, epithelial barrier integrity, and immune homeostasis rather than relying solely on nonspecific microbial elimination. Ecological restoration approaches, including probiotics, prebiotics, synbiotics, OMT, and biofilm-disrupting enzymes or peptides, may promote commensal recolonization and reduce pathogenic biofilm persistence. Pathogen-selective strategies, such as bacteriophage therapy, antimicrobial photodynamic therapy, and CRISPR-Cas based therapeutics, are designed to suppress virulent taxa while preserving the resident microbiota. Host-directed interventions, including NSAIDs, low-dose doxycycline, vitamin D, resolvins/lipoxins, and antioxidants, may attenuate excessive inflammation, matrix degradation, oxidative stress, and bone resorption. Immune-based approaches, such as mucosal vaccination, IgA/IgG enhancement, and immune checkpoint blockade in OSCC, may further improve microbial control and antitumor immunity. Together, these strategies highlight a shift toward mechanism-based restoration of oral microbial balance, controlled inflammation, tissue repair, and oral homeostasis.

Oral diseases reflect host-microbiome-immune disequilibrium, and emerging therapies aim to restore this balance, as summarized in **Figure 4**.

## 6 Toward precision oral medicine

### 6.1 Multi omics integration

Single-omics methods have greatly advanced our understanding of the oral microbiome, but they don’t provide a complete picture of the biology behind oral diseases. For example, amplicon-based profiling can tell us which microbes are present, but it doesn’t give enough details about the differences between strains, their functions, the body’s immune responses, or the effects on metabolism. Bacteria, mouth protective layers, the immune system, and the environment interact to create oral illnesses. Because of this, no single method can explain how microbial imbalances injure tissue. Multi-omics is essential for bringing oral microbiome research beyond observations to deeper, more helpful insights for understanding and treating oral illnesses (192).

Metagenomics identifies and analyzes microorganisms’ kinds and roles, whereas metatranscriptomics records their active genes. These technologies provide more about disease-related microbial activity and environmental changes than amplicon-based approaches (193). Metabolomics, proteomics, and metaproteomics contribute to oral microbiome-host interface functional insights beyond genomic and transcriptome levels. These procedures collect metabolites, proteins, and enzymes that regulate the immune system, harm tissue, and disrupt the environment. This helps us comprehend the microbial-host ecosystem’s function, not only its microorganisms (194). Multi-omics also shows how complex oral illness is by merging microbial, host, and clinical layers (195). These levels are important because they provide different perspectives that work together. One layer tells us, “who is there,” another explains “what they can do,” another shows “what they are doing right now,” and another reveals “what biochemical changes are happening as a result.”.

Personalized oral treatment can benefit from multi-omics. By combining microbial, body, and clinical data, it can identify different diseases instead of treating gum disease, tooth decay, and oral cancer as the same. These methods can help identify diseases, assess risks, diagnose, choose therapies, and evaluate their efficacy (196). Oral conditions are biologically diverse, thus this wide approach is crucial. Gum disease, tooth decay, peri-implant disease, and oral cancer differ in bacteria, body reaction, tissue damage, and treatment. Multi-omics helps uncover disease subgroups beyond microorganisms. Long-term host-microbiome research have revealed that the immune system and microbial activity determine gum disease progression, emphasizing the need of combining host and microbiome data across time (197).

Califf et al. applied a meta-omics workflow combining 16S rRNA sequencing, shotgun metagenomics, and tandem mass spectrometry to subgingival and supragingival biofilms from adults with chronic periodontitis before and after treatment. Samples were collected from 3–12 mm periodontal pockets at baseline, 2 weeks, and 3 months after treatment. Metabolite diversity was significantly correlated with maximum pocket depth at baseline (rho = 0.21, P = 0.008), and patients who did not improve showed greater taxonomic instability than responders (UniFrac distance: t = −3.59, P = 0.002), whereas metabolic profiles showed the opposite pattern (Bray-Curtis: t = 2.42, P = 0.02) (198). These findings suggest that multi-omics, particularly metabolomics, may help predict periodontal treatment response and provide informative inputs for future AI-based clinical models. Another study combined 5-region 16S rRNA sequencing with whole-exome sequencing in OSCC tissues and validated key findings using fluorescence *in situ* hybridization, showing that intratumoral bacterial features were associated with somatic mutational signatures and short-term disease-free survival (199). Feher et al. used a Random Forest model to integrate demographic, clinical, microbiological, and treatment-related data from 414 patients and predict individual periodontal treatment responses at 1 year. The model achieved an internal AUROC of 0.93 and an external AUROC of 0.76 in an independent cohort of 78 patients. This study shows how AI can transform complex oral disease information into individualized prediction of treatment outcomes, although prospective validation is still needed (200).

Multi-omics can refine our understanding of oral diseases by integrating microbial, host, and metabolic data. It enables more precise patient classification based on disease progression, inflammation, and treatment response, supporting personalized oral medicine. However, standardized methods and larger longitudinal studies are still needed before multi-omics can be widely applied in diagnosis and treatment.

### 6.2 AI and biomarker discovery

Conventional statistical methods may miss oral disease’s nonlinear interactions when oral microbiome datasets become more high-dimensional, sparse, compositional, and multimodal. Machine learning and deep learning provide important tools for extracting clinically relevant signals from complex oral datasets.

AI provides powerful tools for microbiome research by helping identify complex patterns and associations within high-dimensional microbial data (201, 202). Consequently, models that integrate microbiome features with imaging, histopathology, transcriptomics, or clinical variables may offer substantially greater predictive value than any single modality alone. Multimodal AI frameworks could potentially be used to identify early malignant transformation in high-risk lesions, predict tumor aggressiveness, or estimate immunotherapy responsiveness by linking microbial patterns with tumor-immune states (203, 204).

AI in oral microbiome research should not be evaluated solely on classification accuracy. A high area under the curve (AUC) is insufficient if models lack calibration, interpretability, or generalizability across populations. Oral microbiome data are susceptible to domain shift; subsequent models must be assessed for robustness, external validity, and biological plausibility (205). Another major area of research is using AI for long-term and tailored predictions (206). Because oral diseases fluctuate over time, AI models that track changes in the microbiome and host response over time may be more useful than cross-sectional models.

However, robust classification performance alone is inadequate for clinical translation. In oral microbiome research, a model may exhibit significant discrimination while being clinically unreliable if it is inadequately calibrated, challenging to understand, or unstable across diverse external populations. This worry is especially important because oral microbiome datasets are very sensitive to domain shift caused by changes in geography, food, oral niche sampling, sequencing methods, and patient characteristics. Consequently, forthcoming AI models must be evaluated not only for discrimination but also for calibration, robustness, interpretability, biological plausibility, and external validity. Additionally, as oral diseases change over time instead of staying the same, longitudinal modeling may be more useful for accurately capturing progression, instability, and treatment response than just cross-sectional prediction (207).

Practically, the essential question is how AI can improve dental care, not whether it can classify disorders. AI may help with risk assessment, early identification, disease subtyping, treatment selection, and post-treatment monitoring in precision oral medicine. AI integrates microbiome, body, and clinical context data to provide faster, more educated, and tailored therapy. AI may improve decision-making from simple observations to more informed, predictive, and responsive therapies, not replace doctors.

AI systems need more than external validation to be useful in dental treatment. They must deliver clinician-interpretable outputs, clear action steps, and compatibility with screening, diagnostic, and follow-up procedures.

### 6.3 Challenges and future directions

Although oral microbiome research is progressing rapidly, there are still several major challenges preventing it from being more useful in clinical practice. These challenges aren’t just related to technology; they also involve biological, translation, and implementation issues. Together, these challenges define the key goals for the next stage of research.

#### 6.3.1 Biological and methodological complexity

A major challenge in oral microbiome research is the complex mix of biological and research-related factors. The types of microbes present don’t always match up with their activity, and taxonomic detection alone does not fully explain disease heterogeneity, progression, or treatment responsiveness. At the same time, variation in sampling sites, storage conditions, sequencing platforms, and analytical pipelines limits comparability and reproducibility across studies. Figuring out cause and effect is especially tough because the changes in microbes could be either causes of the disease, results of it, or both, depending on the timing and environment. These issues highlight the need for more standardized, long-term studies that focus on understanding the underlying mechanisms.

#### 6.3.2 Translational bottlenecks in clinical validation

While the field has identified many microbial markers and potential biomarkers, turning these findings into practical clinical tools is still a challenge. Most of the current research consists of short, one-time studies that help form ideas but aren’t enough for actual treatments. There’s a lack of large, long-term studies and trials that can test whether microbiome-based or host-microbiome methods can improve diagnosis, prediction, or treatment. Without these next steps, the field may continue to generate biologically informative findings with limited clinical applicability.

#### 6.3.3 Barriers to real-world clinical implementation

Even when microbial or AI-derived signals show promising statistical performance, their integration into routine clinical care often remains unclear. A lot of proposed biomarkers don’t have clear action thresholds, points of integration into workflows, or proof that using them impacts clinical decisions in important ways. Other problems include high costs, uncertainty about payment, regulatory monitoring, explainability requirements, and ethical issues relating to data governance and algorithmic bias. These implementation problems are not ancillary; they are at the heart of whether research on the oral microbiome can go from being a good idea to having a real-world therapeutic effect.

#### 6.3.4 Future directions toward precision oral medicine

In the future, the field has to go beyond the continuing collection of fragmented taxonomic fingerprints and single-center cross-sectional classifiers. Longitudinal, mechanism-oriented, and clinically anchored research frameworks are therefore necessary to elucidate the dynamic interactions among microbial ecology, host immunity, and disease evolution throughout time. In particular, future progress will depend on well-designed longitudinal cohorts, multi-layer host-microbiome profiling, causal and functional validation of candidate pathways, biomarker systems linked to therapeutic decision-making, and workflow-ready precision tools that can be meaningfully integrated into real-world oral care. The forthcoming phase of oral microbiome research should focus on providing a more exact characterization of dysbiosis, while also converting this knowledge into practical biological insights and clinically applicable interventions.

## 7 Discussion

A key discovery from this research is that oral disorders are better understood as systemic failures of an interconnected host-microbiome-immune network rather than as discrete infections caused by individual bacteria. In normal circumstances, the oral cavity sustains ecological and immunological equilibrium via synchronized interactions among epithelial barriers, salivary defenses, innate and adaptive immunity, and CMC. When this balance is broken, illness happens. This lets harmful microorganisms flourish, inflammation become worse, and tissue damage get worse. In this view, gum disease, tooth decay, and oral cancer are better understood as indicators of systemic imbalances within the oral ecology and immune system rather than mere infections.

This method shows how typical models that only look at infections don’t work well when trying to figure out oral disorders. Some bacteria are still vital, but just knowing which ones are there doesn’t necessarily help us understand how a disease starts or how it responds to treatment. The way bacteria interact with each other, how the immune system responds, how well the mouth’s protective barrier works, and the mouth’s local environment all affect how a disease gets worse. It’s vital to remember that the host’s immune response isn’t only a reaction to an imbalance in the microbiome; it’s also an active force that determines whether the microbial communities stay healthy or get sick.

At the molecular level, this active host-microbiome interaction is mediated not merely by microbial imbalance itself, but by a layered communication network. At the molecular level, this active host-microbiome interaction is mediated by a layered communication (208). Microbial-associated molecular patterns, including LPS, lipoproteins, and peptidoglycan fragments, can be recognized by TLR2/4 and NOD1/2, leading to MyD88- or RIP2-dependent activation of NF-κB/MAPK signaling and increased production of TNF-α, IL-6, and pro-IL-1β (209, 210). When additional danger signals such as ROS, extracellular ATP (211), or microbial virulence factors are present, NLRP3 inflammasome activation further promotes caspase-1-mediated maturation of IL-1β and IL-18, thereby amplifying local inflammation and tissue injury (212). In PD, this inflammatory cascade may enhance neutrophil recruitment, matrix metalloproteinase expression, Th17-related responses, and RANKL/OPG-mediated osteoclast activation, ultimately contributing to connective tissue degradation and alveolar bone loss (213).

Microbial metabolites and epithelial barrier signals provide another important regulatory layer. Short-chain fatty acids and indole derivatives can influence Treg differentiation, AhR-IL-22 signaling, antimicrobial peptide production, and epithelial repair, whereas persistent dysbiosis may impair tight-junction proteins such as claudins, occludin, and ZO-1, increasing epithelial permeability and facilitating the translocation of microbial products (214, 215). Therefore, oral dysbiosis represents not only microbial imbalance, but also a disruption of host-microbiome signaling that promotes inflammation, barrier dysfunction, tissue damage, and systemic immune activation.

Together, these signaling pathways support the view that oral dysbiosis should not be regarded as a purely local disturbance, but rather as a potential driver of broader host immune and inflammatory dysregulation. Accumulating evidence indicates that the oral cavity functions as an active immunological and microbial interface capable of influencing systemic health. It can spread harmful microbes, trigger chronic inflammation, change how the immune system works, and communicate with other organs. Connections between oral health and conditions like heart disease, diabetes, digestive issues, brain inflammation, and pregnancy complications show just how important the health of the mouth is for the rest of the body. At the same time, these links reveal a major gap in current research: most studies only show a relationship, not cause-and-effect. While there are plenty of biological signals, we still lack the kind of research that could tell us exactly how to use this information for effective treatments.

These restrictions have clear effects on treatment. If oral disorders are caused by an unstable balance of bacteria and the body’s immune system not working properly, then remedies need to be more than just antibiotics or other general treatments. Instead, they should try to get the mouth back to a healthy equilibrium and make the immune system work right. Traditional treatments are helpful, but they probably won’t work in the long run until the balance of microorganisms and the immune system are fixed. This method suggests that treatments that focus on changing the microbiome, attacking the immune system, and employing more tailored, specific medications might work better.

In the future, we need to stop just collecting random data on microbial species from one-time research in one place. What we truly need are long-term studies that look at how microorganisms, the immune system, and diseases change over time. To achieve genuine progress, we need well-planned, long-term research groups, complete profiles of both the host and the microbiome, and studies that show how these parameters are related to each other. It is also important to make systems for biomarkers that can help doctors decide on therapy and precision tools that can be readily added to daily oral care. The subsequent phase of oral microbiome research should focus on not only enhancing the precision of imbalance identification but also translating these discoveries into actionable insights that facilitate improved treatments.

To better contextualize the current evidence and its clinical translation, we summarize key evidence limitations in **Box 1** and major translational challenges in **Box 2**.

Box 1 **Evidence strength and limitations of the reviewed literature**

The evidence reviewed in this article is heterogeneous and varies substantially in methodological rigor. Different study designs therefore require different levels of interpretation.

Clinical interventional evidence: Randomized or controlled human studies support selected adjunctive periodontal therapies and immune checkpoint inhibitors in recurrent/metastatic HNSCC. However, direct microbiome-stratified interventional evidence in oral disease remains limited.

Systematic reviews and meta-analyses: These studies summarize clinical trends, particularly for probiotics and periodontal adjunctive therapies, but are often limited by heterogeneity in study design, microbial strains, treatment duration, and clinical endpoints.

Observational microbiome studies: Cross-sectional and exploratory microbiome studies identify disease-associated microbial signatures, but they generally cannot determine causality, temporal order, or treatment relevance.

Preclinical mechanistic studies: Animal and *in vitro* models provide mechanistic support for OMT, bacteriophage therapy, CRISPR-based antimicrobials, RvE1, and Treg induction, but these findings require further human validation.

Interpretation: Exploratory, associative, preclinical, and clinically validated findings should be distinguished throughout the manuscript to avoid overinterpretation of observational or model-based data.

Box 2 **Current limitations and translational challenges of microbiome-based therapies**

Although microbiome-based therapies offer promising opportunities to restore host-microbiome-immune balance, several limitations still restrict their clinical translation. Safety remains a major concern, particularly for live microbial products, which may carry risks of pathogen transmission, invasive infection in vulnerable patients, antimicrobial-resistance transfer, or unintended ecological disturbance. Regulatory pathways for probiotics, live biotherapeutic products, MT, and engineered microbial therapies are also still evolving, creating uncertainty in product classification, quality control, and clinical approval. In addition, clinical implementation is limited by challenges in defining active ingredients and functional potency, ensuring batch-to-batch consistency and stability, selecting suitable patients, achieving durable engraftment, and choosing appropriate delivery routes. Long-term effects, including persistent colonization and metabolic or immune spillover, remain insufficiently understood. Future translation will require rigorous donor or strain screening, standardized manufacturing and quality-control criteria, biomarker-guided patient stratification, early regulatory engagement, and long-term safety monitoring.

## 8 Conclusion

Ultimately, oral diseases are changes in the balance between the host, microbiota, and immune system that depend on the situation, not just infections. This systems-level perspective elucidates the variations in diseases and their duration, as well as the constraints of microbial-only therapies. In the future, oral medicine will focus more on restoring ecosystems, recalibrating the immune system, and making precise treatments based on integrated biological data than on getting rid of random microorganisms. To move research forward, it has to progress from descriptive dysbiosis to mechanism-based frameworks for prediction, prevention, and therapy that are useful for treatment. AI will help precision medicine by making personalized treatments better.

## Supplementary Appendix S1. Evidence Mapping and Reference Selection Rationale

1. Purpose of this appendix.

The main manuscript describes the literature search strategy, databases, search terms, and general selection criteria. This supplementary appendix further clarifies how the selected references were organized and used to support the conceptual framework of host–microbiome–immune disequilibrium in oral disease.

2. Evidence domains.

The selected references were grouped according to their contribution to the review framework:

1. Oral microbiome ecology and biofilm organization.

These studies were used to describe the healthy oral microbiome, microbial community structure, biofilm formation, commensal functions, metabolic cross-feeding, pH balance, and redox regulation.

2. Oral mucosal immunity and host defense.

These references supported the discussion of epithelial barrier function, salivary defense, antimicrobial peptides, neutrophils, macrophages, dendritic cells, T cells, B cells, and sIgA-mediated immune surveillance.

3. Disease-specific dysbiosis mechanisms.

Studies in this group were used to explain dysbiosis in periodontitis, dental caries, and OSCC, including inflammation-stabilized anaerobic dysbiosis, sugar-driven acidification, biofilm remodeling, epithelial disruption, immune dysregulation, and tumor-associated microbial remodeling.

4. Oral-systemic inflammatory links.

These references were included when they directly supported the relationship between oral dysbiosis and systemic inflammatory conditions, including cardiovascular, metabolic, gastrointestinal, neurological, renal, and pregnancy-related disorders.

5. Therapeutic recalibration of the oral ecosystem.

This group included studies on probiotics, prebiotics, oral microbiome transplantation, biofilm-disrupting strategies, bacteriophage therapy, antimicrobial photodynamic therapy, CRISPR-based approaches, host-modulatory therapies, pro-resolving mediators, and immunotherapy-related strategies.

6. Multi-omics, AI, and precision oral medicine.

These studies were selected when they addressed biomarker discovery, disease stratification, treatment-response prediction, multi-omics integration, microbiome-based diagnostics, or artificial intelligence-assisted clinical decision support.

3. Reference selection rationale.

References were selected not only based on publication date or citation frequency, but also according to their relevance to the central framework of this review. Priority was given to studies with one or more of the following values:

1. Conceptual value: helping define oral diseases as disruptions of host-microbiome-immune balance rather than single-pathogen infections.
2. Mechanistic value: explaining microbial metabolism, epithelial barrier dysfunction, inflammatory signaling, immune-cell activation, or biofilm remodeling.
3. Clinical value: linking microbial or immune alterations to disease progression, clinical phenotypes, or treatment response.
4. Translational value: supporting therapeutic development, biomarker discovery, precision medicine, multi-omics integration, or AI-assisted applications.

4. Evidence synthesis approach

Because the included studies varied in design, model system, disease context, and clinical maturity, the evidence was narratively synthesized rather than quantitatively pooled. Greater weight was given to findings that were mechanistically plausible, clinically relevant, consistent with existing evidence, and supported by complementary study types.

This evidence-mapping approach allowed the review to integrate microbiome ecology, mucosal immunity, disease mechanisms, therapeutic strategies, and emerging precision-medicine technologies while maintaining focus on the central concept of host-microbiome-immune disequilibrium in oral disease.

## Glossary

ADI: Arginine Deiminase
AI: Artificial Intelligence
AMPs: Antimicrobial Peptides
AMR: Antimicrobial Resistance
aPDT: Antimicrobial Photodynamic Therapy
AUC: Area Under the Curve
CBCT: Cone Beam Computed Tomography
CFU: Colony-Forming Units
CMC: Commensal Microbial Communities
COX: Cyclooxygenase
CRISPR-Cas: Clustered Regularly Interspaced Short Palindromic Repeats–CRISPR-associated proteins
CRP: C-Reactive Protein
DCs: Dendritic Cells
ERK: Extracellular Signal-Regulated Kinase
GCF: Gingival Crevicular Fluid
HNSCC: Head and Neck Squamous Cell Carcinoma
IBD: Inflammatory Bowel Disease
ICIs: Immune Checkpoint Inhibitors
IgA: Immunoglobulin A
IgG: Immunoglobulin G
IL-10: Interleukin-10
IL-17: Interleukin-17
IL-1β: Interleukin-1 Beta
IL-6: Interleukin-6
IL-8: Interleukin-8
LDD: Low-Dose Doxycycline
LPS: Lipopolysaccharide
MAPK: Mitogen-Activated Protein Kinase
MMP-8: Matrix Metalloproteinase-8
MMPs: Matrix Metalloproteinases
MOI: Multi Omics Integration
MPO: Myeloperoxidase
MRI: Magnetic Resonance Imaging
MT: Microbiome Transplantation
NETs: Neutrophil Extracellular Traps
NF-κB: Nuclear Factor Kappa-B
NSAIDs: Nonsteroidal Anti-Inflammatory Drugs
OL: Oral Leukoplakia
OLP: Oral Lichen Planus
OMT: Oral Microbiome Transplantation
OSCC: Oral Squamous Cell Carcinoma
PD: Periodontal Disease
PD-1: Programmed Cell Death Protein 1
PD-L1: Programmed Death-Ligand 1
PGE2: Prostaglandin E2
PI3-Akt: Phosphoinositide 3-Kinase-Protein Kinase B
PNAG: Poly-N-acetylglucosamine
PRRs: Pattern Recognition Receptors
RANKL: Receptor Activator of Nuclear Factor Kappa-B Ligand
ROS: Reactive Oxygen Species
RvE1: Resolvin E1
SDD: Sub-antimicrobial Dose Doxycycline
sIgA: Secretory Immunoglobulin A
SRP: Scaling and Root Planing
STAT3: Signal Transducer and Activator of Transcription 3
TMJ: Temporomandibular Joint
Th17: T Helper 17 Cells
TLRs: Toll-like Receptors
TNF-α: Tumor Necrosis Factor Alpha
Tregs: Regulatory T Cells
TREM2: Triggering Receptor Expressed on Myeloid Cells 2
VDS: Vitamin D Supplementation.
